# A Novel 3-*O*-rhamnoside: 2″-*O*-xylosyltransferase Responsible for Terminal Modification of Prenylflavonol Glycosides in *Epimedium pubescens* Maxim.

**DOI:** 10.3390/ijms232416050

**Published:** 2022-12-16

**Authors:** Yu Yao, Jiajun Gu, Yanjiao Luo, Yixin Zhang, Yuanyue Wang, Yongzhen Pang, Shangang Jia, Chaoqun Xu, Doudou Li, Fengmei Suo, Guoan Shen, Baolin Guo

**Affiliations:** 1 Key Laboratory of Bioactive Substances and Resources Utilization of Chinese Herbal Medicines, Ministry of Education, Institute of Medicinal Plant Development, Chinese Academy of Medical Sciences, Peking Union Medical College, Beijing 100193, China; 2 School of Chinese Materia Medica, Tianjin University of Traditional Chinese Medicine, Tianjin 301617, China; 3 Institute of Animal Sciences, The Chinese Academy of Agricultural Sciences, Beijing 100193, China; 4 College of Grassland Science and Technology, China Agricultural University, Beijing 100193, China

**Keywords:** *Epimedium pubescens* Maxim., icariin, prenylflavonol, UDP-glycosyltransferase, xylosyltransferase

## Abstract

Prenylated flavonol glycosides in *Epimedium* plants, as key medicinal components, are known to have great pharmaceutical activities for human health. Among the main prenylated flavonol glycosides, the modification mechanism of different sugar moieties is still not well understood. In the current study, a novel prenylated flavonol rhamnoside xylosyltransferase gene (*EpF3R2″XylT*) was cloned from *E. pubescens*, and the enzymatic activity of its decoding proteins was examined in vitro with different prenylated flavonol rhamnoside substrates and different 3-*O*-monosaccharide moieties. Furthermore, the functional and structural domains of EpF3R2″XylT were analyzed by bioinformatic approaches and 3-D protein structure remodeling. In summary, *EpF3R2″XylT* was shown to cluster with GGT (glycosyltransferase that glycosylates sugar moieties of glycosides) through phylogenetic analysis. In enzymatic analysis, *EpF3R2″XylT* was proven to transfer xylose moiety from UDP-xylose to prenylated flavonol rhamnoside at the 2″-OH position of rhamnose. The analysis of enzymatic kinetics showed that EpF3R2″XylT had the highest substrate affinity toward icariin with the lowest *K_m_* value of 75.96 ± 11.91 mM. Transient expression of *EpF3R2″XylT* in tobacco leaf showed functional production of EpF3R2″XylT proteins in planta. *EpF3R2″XylT* was preferably expressed in the leaves of *E. pubescens*, which is consistent with the accumulation levels of major prenylflavonol 3-*O*-triglycoside. The discovery of EpF3R2″XylT will provide an economical and efficient alternative way to produce prenylated flavonol trisaccharides through the biosynthetic approach.

## 1. Introduction

**Figure 1:**
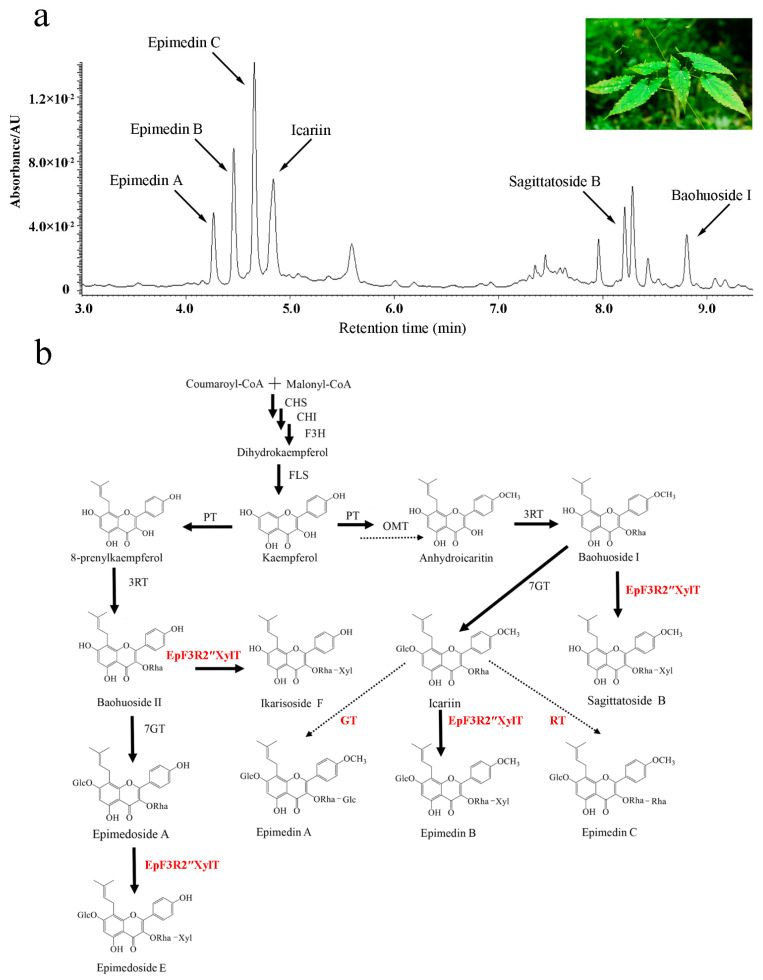
The UPLC profile and proposed biosynthetic pathways of prenylated flavonoids of *Epimedium pubescens* Maxim. (**a**) UPLC profile of extract of *E. pubescens* leaves. (**b**) The proposed biosynthetic pathways of prenylated flavonoids of genus *Epimedium.* Glc: Glucosyl-moiety, Rha: Rhamnosyl-moiety, Xyl: Xylosyl-moiety. PT: Prenyltransferase, OMT: Omethyltransferase, GT: Glucosyltransferase, RT: Rhamnosyltransferase. The dotted arrows show the hypothetical enzymes, the solid arrows show the identified enzymes. The enzymes newly identified in this study are highlighted in red.

As an important traditional Chinese medicine [1], Herba Epimedii (leaves from several species of *Epimedium* L.) possesses multiple pharmaceutical activities such as sexual function promotion, anti-inflammatory, anti-osteoporotic, anti-rheumatic, anti-oxidant, anti-cancer, and anti-angiogenic activity [1,2]. The major pharmacologically active compounds have been shown to be prenylated flavonol glycosides that have multiple sugar moieties at both the 3-OH and 7-OH positions [3,4]. Among these prenylated flavonol glycosides, icariin, epimedin A, epimedin B, and epimedin C are the most predominant compounds with different forms of sugar moieties in *Epimedium* plants [3,4] (Figure 1a). The formation of different disaccharide O-linked modifications at 3-OH among these compounds is not well understood (Figure 1b).

Currently, the availability of prenylated flavonol glycosides remains limited due to the low yield of *Epimedium* plants [5]. The new approaches of synthetic biology may provide an alternative way to produce prenylated flavonol glycosides for medicinal use, which is largely dependent on the elucidation of the biosynthetic pathway of these compounds. To date, a few glycosyltransferase genes have been identified and cloned from the genus *Epimedium* and were proven to only transfer a sugar moiety to either the 3-OH or 7-OH position of prenylated flavonols to form prenylated flavonol glycosides [6,7,8,9,10,11]. Based on the previous studies in the genus *Epimedium* [6,7,8,9], we assumed the detailed scheme of prenylflavonoid glycoside biosynthesis (Figure 1b). Although a few glycosyltransferase (GT) genes were reported in different species of the *Epimedium* genus, the critical GT genes that are responsible for transferring an additional sugar moiety to the rhamnose moiety at 3-OH of icariin to form epimedin A, epimedin B, or epimedin C remain to be determined [7,8]. Therefore, the large-scale production of epimedin A, epimedin B, and epimedin C via synthetic biology approaches has been largely hindered due to limited knowledge of the key biosynthetic genes of prenylated flavonol GTs.

GT genes are able to transfer a sugar moiety to different acceptors and catalyze a key modification of secondary metabolites, leading to dramatic changes in chemical properties, bioactivity, and accessibility [12,13]. Previously, the reported flavonoid GGT (glycosyltransferase that glycosylates sugar moieties of glycosides) was shown to transfer glucose moieties to glycosides and mainly recognize the glucose group of flavonol glucosides [14,15,16]. Furthermore, a few flavonoid glycoside xylosetransferase have been identified previously [17,18]. These previous studies provided the foundation to further identify the novel GGT genes. However, the GGTs that could transfer xylose to 3-*O*-rhamnosides remain less explored.

In this study, we identified a novel xylosyltransferase gene (designated as *EpF3R2″XylT*) from a representative species of the *Epimedium* genus, *E. pubescens*. The recombinant EpF3R2″XylT proteins possessed the catalytic activity of 2″-*O*-xylosylation on prenylflavonol 3-*O*-rhamnoside, namely icariin, baohuoside II, baohuoside I, and epimedoside A, but not flavonol glycosides without C-8 prenylation or prenylated flavonols without 3-*O*-rhamnosylation. Phylogenetic analysis showed that EpF3R2″XylT was closely clustered together with reported flavonoid glycoside GGTs. Our results will provide a foundation to unravel the biosynthetic pathway of prenylflavonol glycosides in *E. pubescens* and develop appropriate synthetic biology approaches to produce these important compounds that are beneficial for human health.

## 2. Results

### 2.1. Identification of EpF3R2″XylT from E. pubescens

**Figure 2:**
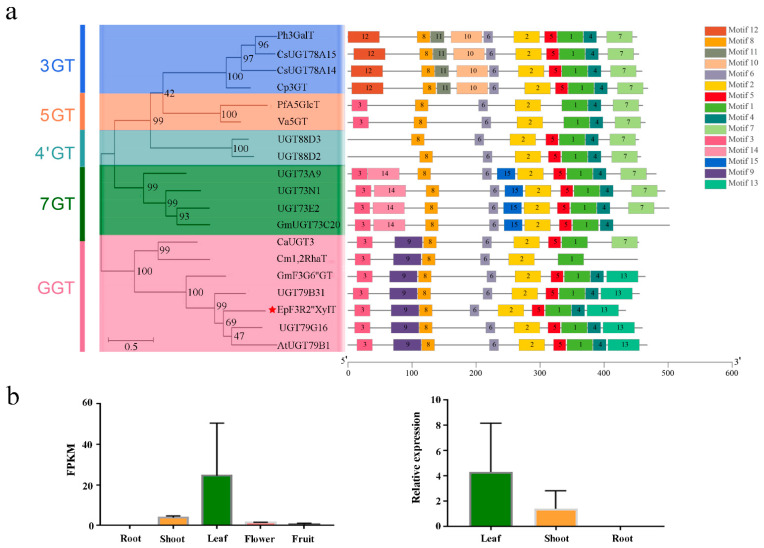
Phylogenetic analysis, protein motif, and expression profile of EpF3R2″XylT. (**a**) The phylogenetic tree and conserved motifs composition was analyzed based on EpF3R2″XylT and reported plant flavonoid GTs; EpF3R2″XylT is displayed with a red star, motifs of numbers 1–15 are shown in different colored domains. (**b**) Expression profile of *EpF3R2″XylT* in different tissues. On the left are the expression profiles including fresh roots, shoots, mature leaves, flowers in full bloom, and fully developed fruits of *E. pubescens* plants from RNA-seq. Two biological replicates for various tissues were prepared, and data are mean values with SD (*n* = 2). On the right is expression analysis of fresh roots, shoots, and mature leaves of *E. pubescens* by quantitative real-time PCR analysis. Data were normalized to actin genes, data are mean values with SD (*n* = 3).

There is a broad spectrum of prenyl flavonoid glycosides in *Epimedium* plants, but varieties of enzymes responsible for the glycosylation of prenylflavonols have not been fully elucidated yet. In the present study, a novel prenylflavonol glycosyltransferase (GT) named *EpF3R2″XylT* was identified from the *E. pubescens* genome. *EpF3R2″XylT* had an ORF of 1299 bp encoding a protein of 433 amino acids and possessed a conserved PSPG domain (the plant’s secondary product glycosyltransferase domain), which acts as the binding site of UDP-sugar (Figure S1). Furthermore, a phylogenetic tree was constructed with EpF3R2″XylT and 17 other reported GT proteins (Figure 2a and Table S1). The results showed that flavonoid GT proteins could generally be divided into five clades including 3GT, 5GT, 4’GT, 7GT, and GGT based on their specific catalytic activity on 3-OH, 5-OH, 4’-OH. 7-OH, or sugar moieties [19]. EpF3R2″XylT was grouped into the GGT clade and was most closely related to AtUGT79B1 from *Arabidopsis thaliana*, which recognized 3-*O*-glucosylated anthocyanidins/flavonols and UDP-xylose [18], suggesting that EpF3R2″XylT might specifically recognize UDP-xylose. EpF3R2″XylT was also closely related to UGT79B31 from *Petunia hybrida* [16] and UGT79G16 from *Ipomoea purpurea* [20], which are GGTs to catalyze the addition of monosaccharide at the C-2″ position of flavonoid-3-*O*-glucoside.

To further determine the conserved structure of EpF3R2″XylT, the flavonoid GT protein sequences were used to identify 15 conservative motifs on the MEME web server (Figure 2a). Most GTs consist of the following motifs with the order of 12-8-11-10-6-2-5-1-4-7-3-14-15-9-13. The PSPG domain (WAPQ-VL-H-SVG-FVTHCGWN-SVLES-GVPMI-P-GDQ) was comprised of motifs 1 and 3 with minor variations among different GT proteins. Moreover, the GT proteins within the same group had similar conserved motifs. For example, motifs 10, 11, and 12 are unique to the 3GT group, while motifs 14 and 15 are unique to the 7GT group; the GGT group mostly has motifs 9 and 13 but lacks motif 7 found in all other groups, which may partially explain the catalytic activity difference for GT proteins. EpF3R2″XylT shared the same motifs as that of the GGT group, indicating that EpF3R2″XylT may possess the capability to add the monosaccharide onto flavonoid glycoside.

In the analysis of tissue expression profile, *EpF3R2″XylT* was shown to be most highly expressed in *Epimedium* leaves (Figure 2b), where the active medicinal compounds prenylflavonol diglycoside and triglycoside mainly accumulated [4]. The full-length sequence of EpF3R2″XylT (Figure S2) was amplified with nested PCR amplification (Table S2) from the cDNA of *E. pubescens*, which was reverse-transcribed from extracted RNA. The obtained full-length sequence was verified by Sanger sequencing.

### 2.2. Enzyme Characterization of EpF3R2″XylT In Vitro

**Figure 3:**
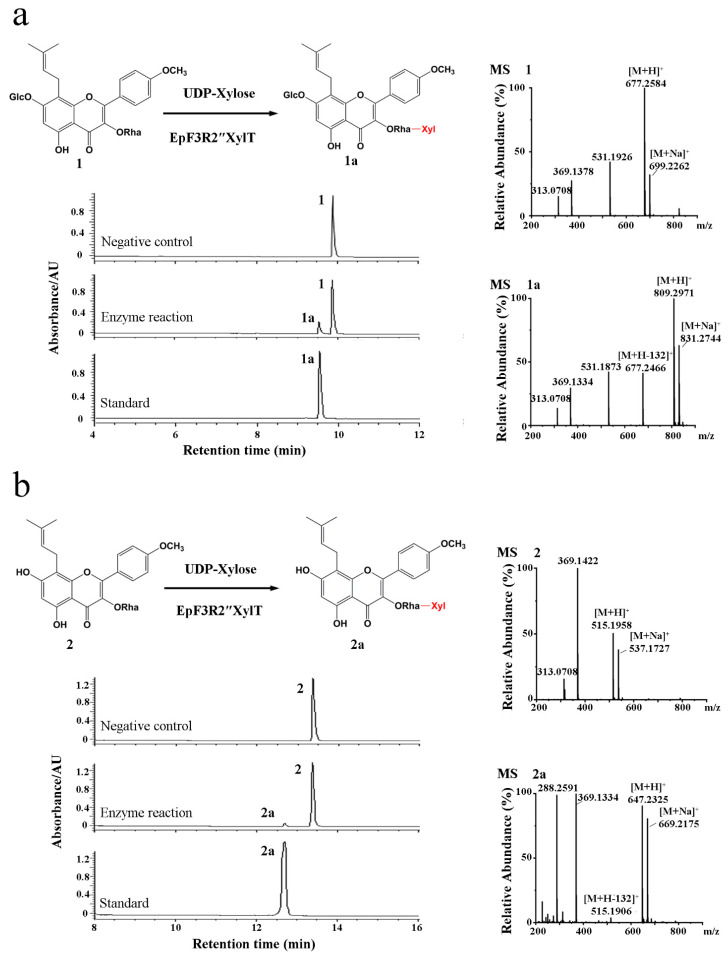
Enzymatic assay of recombinant EpF3R2″XylT with icariin (**1**) and baohuoside I (**2**). (**a**) UPLC/ESI-MS analysis of the product of enzymatic reaction of EpF3R2″XylT with **1**. (**b**) UPLC/ESI-MS analysis of the enzymatic reaction product with **2**.

**Figure 4:**
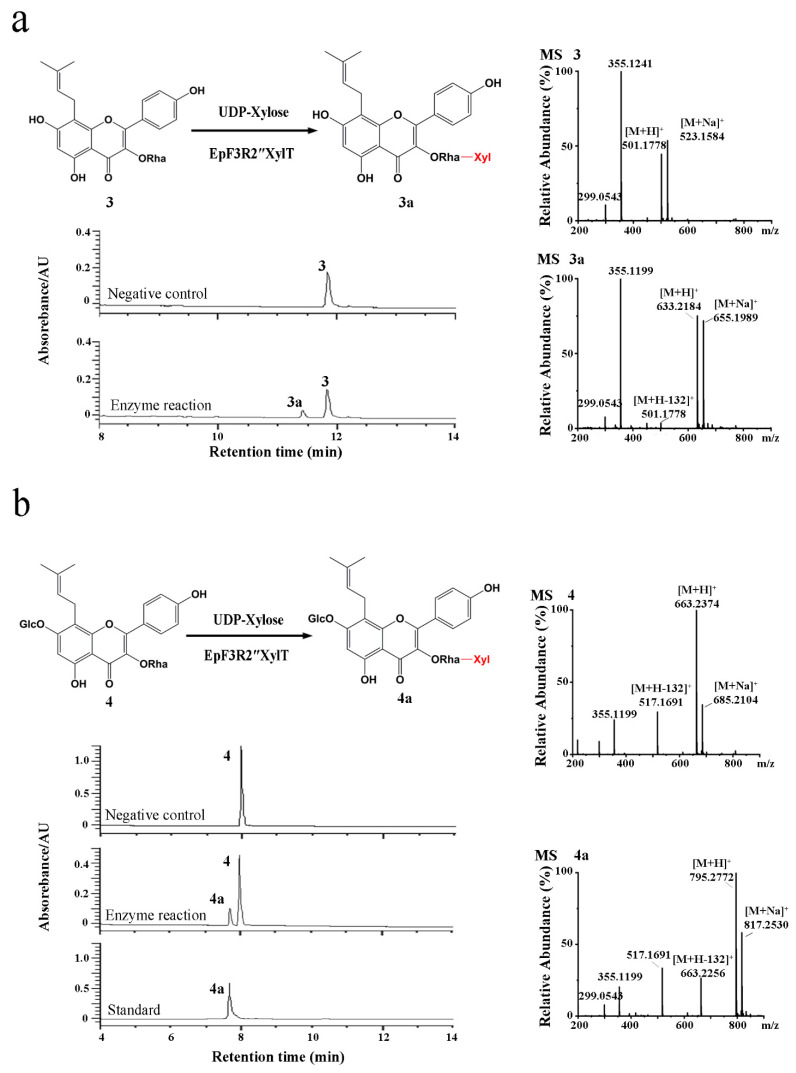
Enzymatic assay of recombinant EpF3R2″XylT with baohuoside II (**3**) and epimedoside A (**4**). (**a**) UPLC/ESI-MS analysis of the enzymatic reaction product of EpF3R2″XylT with **3**. (**b**) UPLC/ESI-MS analysis of the enzymatic reaction product with **4**.

The cloned EpF3R2″XylT was cloned in frame with the maltose-binding protein (MBP) domain (approximately 42.50 kDa) and then introduced into the pMAL-c2X expression vector for the recombinant protein expression in *Escherichia coli* BL21 (DE3). *EpF3R2″XylT* was predicted to encode 433 amino acids with a predicted molecular weight of 48.97 kDa. The purified recombinant proteins fused with the MBP domain showed a molecular weight of approximately 90 kDa (Figure S3). To test the enzyme activity and substrate specificity, the catalytic activities of recombinant EpF3R2″XylT proteins were evaluated with 22 different flavonoid compounds including flavonols, flavonol glycosides, and 8-prenylflavonol glycosides (Table S3 and Figure S4), which were thought to be possible substrates based on the metabolite profiles in *E. pubescens*. A variety of monosaccharide donors including UDP-glucose, UDP-rhamnose, and UDP-xylose have been tested. A negative control was employed with recombinant EpF3R2″XylT proteins deactivated in boiling water for 10 min. UPLC analysis (Figure 3 and Figure 4) indicated that the recombinant proteins have enzymatic activity towards icariin (**1**), baohuoside I (**2**), baohuoside II (**3**), and epimedoside A (**4**) with UDP-xylose as a monosaccharide donor. The above reaction products **1a** and **2a** converted from icariin (**1**) and baohuoside I (**2**) as substrates were identified as epimedin B (**1a**) and sagittatoside B (**2a**) through comparison with authentic standards (Figure 3), respectively. LC–MS spectra were used to further confirm the reaction products (Table S4 and Figure S5). In particular, the only acceptable donor is UDP-xylose, rather than UDP-glucose or UDP-rhamnose, implying that EpF3R2″XylT is a specific xylosyltransferase. Based on the result of LC–MS spectra, the product **3a** of an enzyme reaction with baohuoside II (**3**) and UDP-xylose was presumed to be ikarisoside F (Figure 4, Tables S4 and S5); epimedoside E was speculated to be the product **4a** from epimedoside A (**4**) catalyzed by the recombinant proteins with UDP-xylose (Figure 4, Tables S4 and S5). EpF3R2″XylT showed no activities with other UDP-sugar and flavonoid compounds (Figures S6–S8). Epimedin B (**1a**), sagittatoside B (**2a**), ikarisoside F (**3a**), and epimedoside E (**4a**) were prenylated flavonol 3-*O*-[2-*O*-(-xylosyl)]-rhamnoside, and the products of recombinant proteins displayed a retention time consistent with those of authentic proteins. 

To verify the chemical structure of products of the EpF3R2″XylT catalyzed reaction using icariin as substrate, the reaction products were further separated with a preparative liquid chromatograph and determined by NMR spectroscopy. In the ^1^H NMR spectrum, the appearance of a proton at δ_H_ 4.22 indicated the introduction of a xylose moiety compared to ^1^H NMR spectrum of icariin [21]. The observation of H-2″ (δ_H_ 4.09) in the ^1^H NMR spectrum compared to that of icariin (δ_H_ 5.29) [21] and the observation of C-2″ (δ_C_ 81.21) in the ^13^C NMR spectrum compared to that of icariin (δ_C_ 69.66) [21] suggested that the xylose moiety was introduced to 2″-OH of rhamnose (Figure S9). HSQC and NOESY also confirm this conclusion. The ^1^H-^13^C HSQC spectrum (Figure S10) showed cross-peaks between H-2″ (δ_H_ 4.09) and C-2″ (δ_C_ 81.21), and ^1^H-^1^H NOESY (Figure S11) showed cross-peaks between H-2″ (δ_H_ 4.09) and H-1⁗ (δ_H_ 4.22). These results established that the xylose moiety is connected to the 2′′-OH of rhamnose. All of the data are in agreement with the previously reported data for epimedin B [22]. Consequently, EpF3R2″XylT was able to regiospecifically transfer an additional xylose moiety to the rhamnose group of prenylflavonol 3-*O*-rhamnosides at the 2″ position of rhamnose.

### 2.3. Enzymatic Kinetic Analysis of Recombinant EpF3R2″XylT

**Figure 5:**
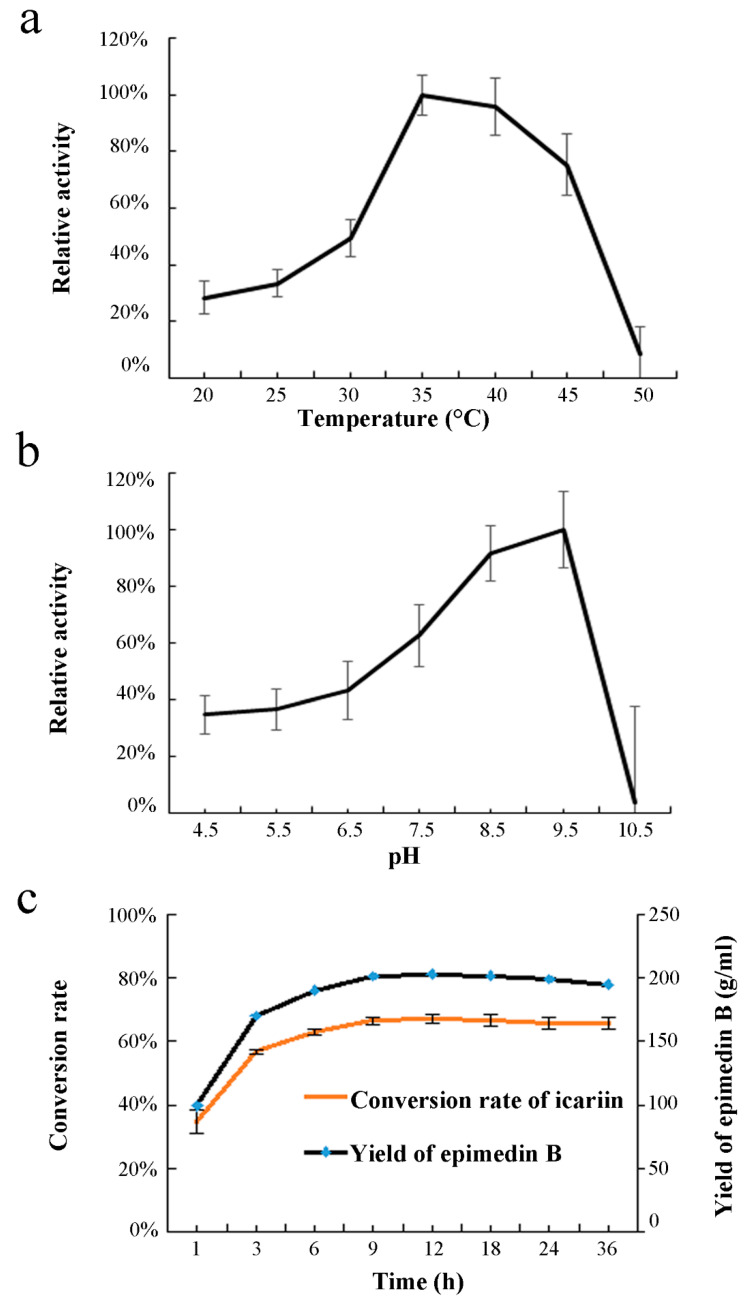
Effects of temperature (**a**), pH (**b**), and time course (**c**) on enzyme activity of EpF3R2″XylT. UDP-xylose and icariin were used as sugar donor and acceptor. Data are mean values with SD (*n* = 3).

The optimal reaction temperature and Ph of recombinant EpF3R2″XylT with icariin as a substrate were further investigated (Figure 5a,b). EpF3R2″XylT showed higher enzymatic activities in the range of 35–45 °C and had maximal catalytic activity at 35 °C. The analysis of the optimal reaction pH showed that the maximal enzymatic activity of EpF3R2″XylT was at pH 9.5. When the pH was higher than 9.5, its enzyme activity decreased significantly. Under optimal incubation conditions, 203.22 g·mL^−1^ of epimedin B with a conversion rate of 67.12% was produced after 12 h of incubation with 200 mM icariin in a final volume of 100 mL; beyond 12 h, the yield of epimedin B would not increase or even decrease (Figure 5c).

The kinetic parameters of recombinant EpF3R2″XylT acting on prenylflavonol 3-*O*-rhamnoside were calculated (Table 1); EpF3R2″XylT showed higher affinity on icariin with *K_m_* 75.96 ± 11.91 mM than baohuoside I with *K_m_* 113.15 ± 37.60 mM and baohuoside II with 123.97 ± 16.45 *K_m_*. The enzyme activity of EpF3R2″XylT with epimedoside A was so low that the Michaelis–Menten equation could not be fitted. The *K_cat_*/*K_m_* ratio was the highest for icariin, followed by baohuoside I and baohuoside II. To summarize, icariin was the most favorable substrate, and EpF3R2″XylT should be the critical glycosyltransferase for epimedin B biosynthesis from icariin. The catalytic activities of EpF3R2″XylT against various substrates are consistent with the higher accumulation of epimedin B and lower levels of sagittatoside B and ikarisoside F in *Epimedium* plants (Figure 1a).

### 2.4. Modeling and Docking of EpF3R2″XylT Protein

**Figure 6:**
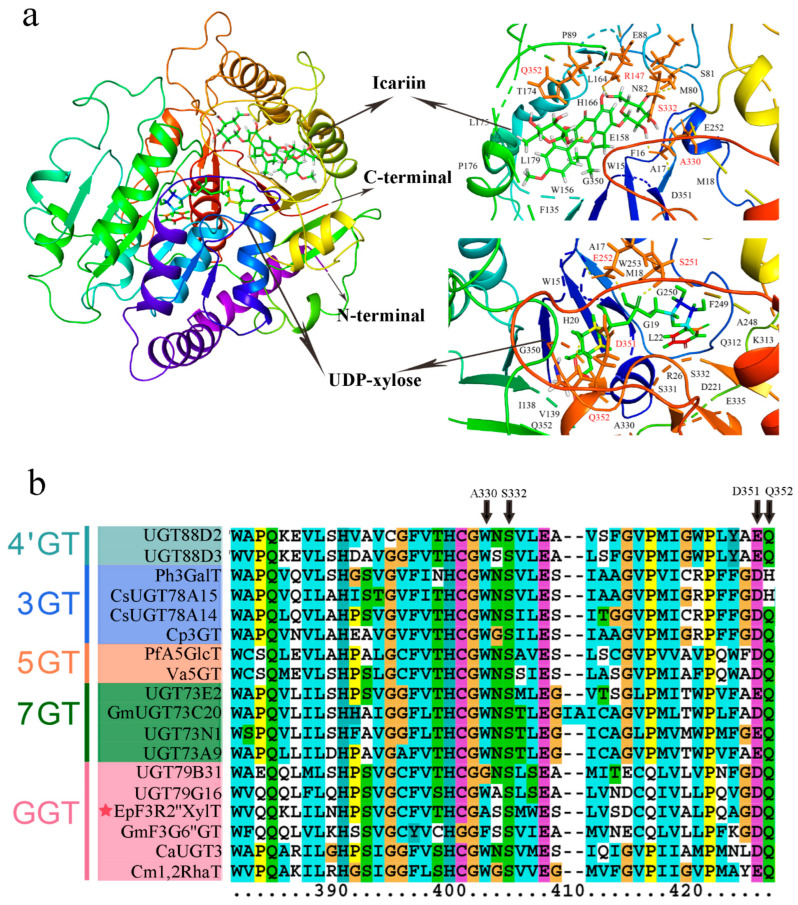
Assumed structural model of EpF3R2″XylT protein and multiple sequence alignment of PSPG domain from EpF3R2″XylT and plant flavonoid GT proteins. (**a**) The structural model of predicted EpF3R2″XylT protein docked with UDP-xylose donor and icariin. The deduced amino acid residues that interacted with UDP-xylose and icariin ligands are highlighted in red. (**b**) Multiple sequence alignment of amino acid sequences of PSPG (the plant secondary product glycosyltransferase) domains from EpF3R2″XylT and other plant flavonoid GTs. EpF3R2″XylT was indicated with a star.

To unravel the catalytic mechanisms of EpF3R2″XylT, the three-dimensional model of EpF3R2″XylT was constructed by homology modeling with the crystal structure of OsGT91C1 (Protein Data Bank code: 7erx.1) as a template, which is known to efficiently catalyze β (1–2) glucosylation [23]. Critical amino acid residues of EpF3R2″XylT were predicted for the possible interactions with both icariin and UDP-xylose (Figure 6a). In EpF3R2″XylT, four amino acid residues (Arg147, Ala330, Ser332, and Gln352) were presumed to contribute to the ligand binding for icariin. Furthermore, Ser251, Glu252, Asp351, and Gln352 were predicted to be critical amino acid sites interacting with UDP-xylose. In addition, the binding sites of substrates were found to be located within a catalytic pocket between the N-terminal and C-terminal domains.

The key amino acids sites interacting with icariin (Ala330, Ser332, and Gln352) and UDP-xylose (Asp351 and Gln352) are generally located within the conserved region in the PSPG domain of EpF3R2″XylT (Figure 6b and Figure S1), indicating these amino acid sites might play a key role in catalyzing the xylosylation of icariin. Ala330 was found only in EpF3R2″XylT, not in other flavonoid glycosyltransferases, suggesting that Ala330 is likely to play a critical role in deciding the specificity of prenylflavonoids; thus, it is worth further investigating the effects of Ala330 in the future.

### 2.5. Transient Expression of EpF3R2″XylT in Tobacco Leave

**Figure 7:**
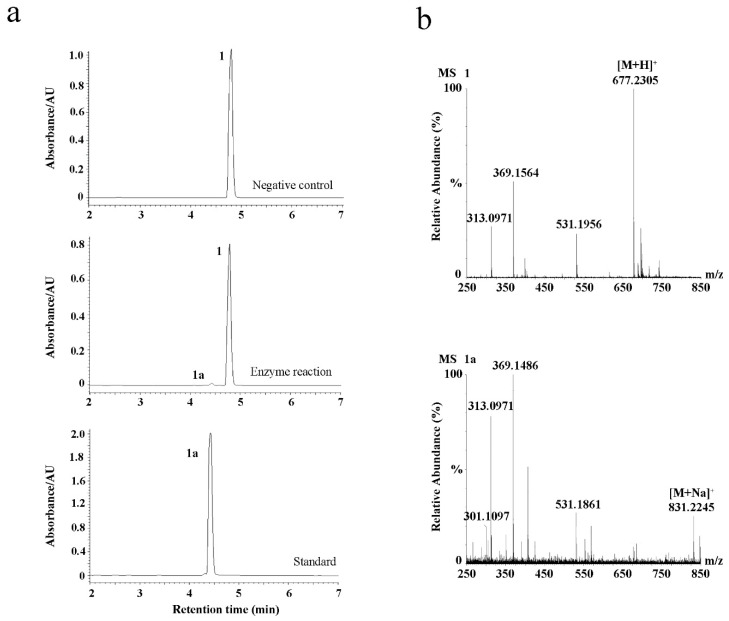
Transient expression of EpF3R2″XylT in tobacco leaves. (**a**) UPLC analysis of negative control, enzymatic reaction of EpF3R2″XylT with 1, and standard of 1a. (**b**) UPLC/ESI-MS analysis of the enzymatic reaction with **1** and **1a**.

To confirm the enzyme activity of EpF3R2″XylT, we transiently expressed *EpF3R2″XylT* in tobacco leaves by *Agrobacterium tumefaciens* infiltration. After 3 days, crude EpF3R2″XylT proteins were prepared from the infected leaves to examine the enzyme activities towards icariin, and a negative control was performed with the empty vector Pk7WG2D.1. UPLC analysis (Figure 7a) showed that crude EpF3R2″XylT proteins incubated with icariin and UDP-xylose yielded a new product peak, which was identified as epimedin B by the retention time and LC-MS (Figure 7b). These results confirmed the enzymatic function of EpF3R2″XylT in planta.

### 2.6. Subcellular Localization of EpF3R2″XylT

**Figure 8:**
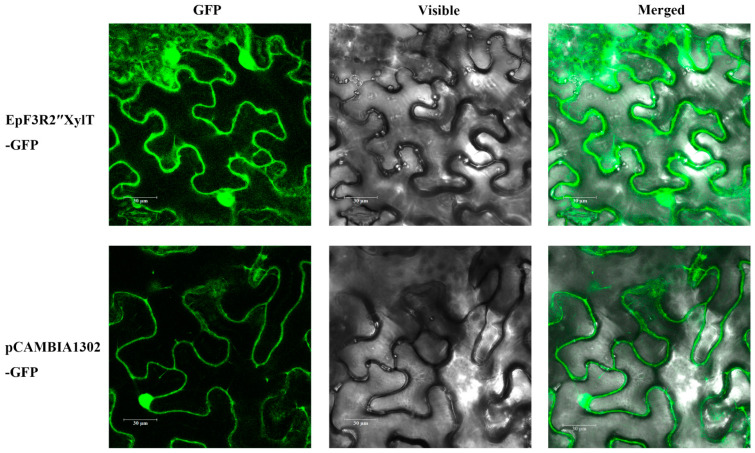
Subcellular localization of EpF3R2″XylT. EpF3R2″XylT-GFP and pCAMBIA1302 vector controls were transferred to *N. benthamiana* leaf cells and observed under confocal laser-scanning microscopy. Scale bars = 30 μm.

We analyzed the subcellular localization of EpF3R2″XylT through transient expression in leaf cells of *N. Benthamiana*. *EpF3R2″XylT* was fused in-frame to *GFP*. The vector control pCAMBIA1302-*GFP* showed typical nuclear and cytoplasmic localization for GFP (Figure 8). The florescent pattern of EpF3R2″XylT-GFP was similar to that of the vector control, indicating that EpF3R2″XylT proteins are targeted to the cytosol. Most plant GTs generally show activities in the cytoplasm, and the glycosylated products are transported through membrane-bound transporter systems that recognize the glycosyl residues [24]. The results of subcellular localization suggested that EpF3R2″XylT should have a normal function similar to other known GTs in the cytoplasm. 

## 3. Discussion

Herba Epimedii is a well-known traditional Chinese medicine capable of stimulating and heightening sexual desire, which is used to effectively treat infertility. The pharmaceutically active compounds have been proven to be a broad spectrum of flavonol glycosides in the genus *Epimedium*. These flavonol glycosides are generally kaempferol derivatives that have been modified by prenylation, methylation, and glycosylation [1,2,3]. The most abundant compounds are icariin, epimedin A, epimedin B, and epimedin C. In particular, they all have a different disaccharide O-linked at 3-OH and a glucose moiety at 7-OH (Figure 1b). The rhamnosyltransferase and glucosyltransferase genes responsible for the 3-*O*-rhamnosylation and 7-*O*-glucosylation of prenylflavonols have been identified in the genus *Epimedium* [6,7,8,9], but the glycosyltransferases remain unknown for the synthesis of disaccharides at 3-OH.

In this study, the cloning and functional characterization of *EpF3R2″XylT* from *E. pubescens* was described, which encodes a prenylflavonol 3-*O*-rhamnoside: 2″-*O*-xylosyltransferase, a key enzyme for the production of epimedin B in *E. pubescens*. The results showed that EpF3R2″XylT only had enzymatic activity toward UDP-xylose as a donor and did not have catalytic activity on UDP-glucose and UDP-rhamnose, implying that EpF3R2″XylT recognized xylose in a regiospecific manner. Other compounds such as epimedin A and epimedin C, which have a different disaccharide O-linked at 3-OH, should be biosynthesized by other unknown glycosyltransferases. *EpF3R2″XylT* is a rarely identified gene responsible for the addition of UDP-xylose to 3-*O*-rhamnoside. According to previous reports, AtUGT79B1 is a xylosyltransferase that recognized flavonoid glycosides from *Arabidopsis thaliana*, which recognizes 3-*O*-glucosylated anthocyanidins/flavonols [18], while F3GGT1 from *Actinidia chinensis* can catalyze the addition of UDP-xylose to cyanidin 3-*O*-galactoside [17]. Combining the UPLC/ESI-MS analysis and NMR spectroscopy of reaction products yielded, the products of enzyme reaction of recombinant EpF3R2″XylT proteins were identified as prenylated flavonol 3-*O*-[2-*O*-(-xylosyl)]-rhamnoside, in which the xylose moiety was introduced to 2″-OH of rhamnose. These results were consistent with the fact that most of the prenylated flavonol triglycosides in the genus *Epimedium* are prenylated flavonol 3-*O*-[2-*O*-(-glycosyl)]-rhamnoside, and only a small number of prenylated flavonol triglycosides are 3-*O*-[4-*O*-(-glycosyl)]-rhamnoside [25].

The in vitro enzyme assay showed that icariin is the most favorable substrate for EpF3R2″XylT with the lowest *K_m_* value (75.96 ± 11.91 mM), which is much lower than the *K_m_* value of CaUGT3 for apigenin 7-*O*-glucoside (140 mM), quercetin 3-*O*-glucoside (380 mM), and kaempferol 3-*O*-glucoside (360 mM) [26], and is also lower than the *K_m_* value of EsGT1 for icariin (362 mM), indicating that *EpF3R2″XylT* is a GGT gene with high affinity. Substrate availability is a determining factor in glycosylation in a cellular context. Except for icariin, EpF3R2″XylT was able to use baohuoside II, baohuoside I, and epimedoside A as substrates to form ikarisoside F, sagittatoside B, and epimedoside E, respectively. These results were consistent with the abundant accumulation of epimedin B (Figure 1a) but low levels of ikarisoside F, sagittatoside B, and epimedoside E in *Epimedium* plants [25], implying that EpF3R2″XylT is a determinant factor for the biosynthesis of these compounds in *E. pubescens*. Nevertheless, with the consideration of a large amount of UGT gene copies in planta, other *EpF3R2″XylT* orthologs cannot be excluded fully, which still need to be further tested in the future.

In addition, the substrates of EpF3R2″XylT such as icariin, baohuoside II, baohuoside I, and epimedoside A, were kaempferol or kaempferide derivatives with both C-8 prenylation and 3-*O*-rhamnosylation (Figure 3 and Figure 4). The presence or absence of 4’-*O*-methyl and 7-*O*-glucosyl of substrates did not affect the recognition of the recombinant enzyme. However, both flavonol glycosides without C-8 prenylation such as kaempferide-3-*O*-rhamnoside and prenylated flavonols without 3-*O*-rhamnosylation such as icariside II could not be catalyzed. These results suggested that both the C-8 prenyl group and 3-*O*-rhamnose are essential for catalyzation. These results also suggested that EpF3R2″XylT acts as the final step for the biosynthesis of prenylated flavonols glycosides in *E. pubescens*. The glycosylation remarkably increases the solubility of prenylated flavonols, which might have correspondingly contributed to the high content of prenylated flavonol glycosides in *E. pubescens*.

In the current study, a xylosyltransferase *EpF3R2″XylT* gene was isolated and functionally characterized from *E. pubescens*, which is able to catalyze the glycosylation of prenylflavonol 3-*O*-rhamnoside. The in vitro enzyme assay explored the optimal incubation condition for forming an important medicinal ingredient of epimedin B via the recombinant enzyme. Thus, our study could promote a significant jump in the efficient production of critical medicinal components of prenylflavonol diglycoside and triglycoside.

## 4. Materials and Methods

### 4.1. Plant Materials and Growth Conditions

Two-year-old seedlings of *Epimedium pubescens* Maxim. Were collected from Le Shan City (29° N, 103° E) in Sichuan Province, China. The seedlings were transferred to flowerpots and grown in the greenhouse of the Beijing Institute of Medicinal Plant Development. Fresh roots, shoots, mature leaves, flowers in full bloom, and fully developed fruits of *E. pubescens* plants were collected and frozen immediately in liquid nitrogen, each with two biological replicates, then stored at −80 °C in a refrigerator for RNA extraction and RNA sequencing [27]. Fresh roots, shoots, and mature leaves of *E. pubescens* plants were collected for quantitative real-time PCR (qRT-PCR), each with three biological replicates.

### 4.2. Profiling Flavonoids in E. pubescens

A measure of 0.2 g of dry leaf powder of *E. pubescens* was weighed and extracted by 20 mL of 50% ethanol with ultrasonic for 30 min at room temperature. The extract was filtered through a 0.22 μm filter for UPLC analysis. The authentic flavonoids were purchased from the Shanghai Yuanye Bio-Technology Co., Ltd., Shanghai, China.

### 4.3. Analysis of the Expression Levels of EpF3R2″XylT

The ABI 7500 real-time detection system (Applied Biosystems, Waltham, MA, USA) was used to perform qRT -PCR with the 2 × RealStar Green Fast Mixture (GeneStar, Shanghai, China). The actin gene was selected from the genome of *E. pubescens* as an internal control. Each reaction was performed in three biological replicates and analyzed by the 2^−ΔΔCT^ method. The primer sequences for qRT -PCR are shown in Table S2.

### 4.4. Cloning and Heterologous Expression of EpF3R2″XylT in Escherichia coli

The total RNA from fresh leaves of *E. pubescens* was extracted by using the Eastep^®^ Super total RNA Extraction Kit (Promega, Shanghai, China) and reverse-transcribed to Cdna with a FastKing One-Step RT-PCR Kit (TIANGEN Biotech, Beijing, China). The coding sequence of *EpF3R2″XylT* was amplified from *E. pubescens* Cdna using Q5^®^ High-Fidelity DNA Polymerases (New England Biolabs, Ipswich, MA, USA) with the first-round primers, then the products of the first round of PCR were subjected to the second round of amplification using the second-round primers (Table S2). The final PCR products were purified by using the AxyPrep DNA Gel Extraction Kit (Corning Inc., Corning, NY, USA) and amplified with the primers with the restriction site (Table S2); the products were cloned into the pMAL-c2X vector (New England Biolabs, MA, USA) by using the LanGene Seamless Cloning Assembly Kit (LANY, Beijing, China). After verification of the sequences by Sanger sequencing, the resulting pMAL-c2X-*EpF3R2″XylT* vectors were transformed into the competent cells of *Escherichia coli* strain BL21 (DE3) (TransGen Biotech, Beijing, China).

The positive bacterial clone BL21 (DE3) harboring the recombinant pMAL-c2X- *EpF3R2″XylT* vector was incubated in Luria-Bertani (LB) media at 37 °C until the OD_600_ reached 0.5. The recombinant proteins were induced by 0.45 mM isopropyl-b-D-thiogalactopyranoside (IPTG). After 24 h of incubation at 16 °C with constant shaking at 100 rpm, the bacterial cells were collected by centrifugation at 4 °C and stored at −80 °C until purified. Maltose-binding protein (MBP) was affinity purified using maltose-binding resin according to the pMAL^™^ Protein Fusion and Purification System (New England Biolabs, Ipswich, MA, USA), and then desalinated and concentrated with a 30 kDa Amicon-Ultra-15 centrifuge filter (Millipore, Burlington, MA, USA). The fusion recombinant EpF3R2″XylT proteins were detected by SDS-PAGE with the Coomassie brilliant blue staining, and the concentration of purified proteins was quantified using the method of Bradford [28] with BSA as a standard.

### 4.5. Enzymatic Assays of Recombinant EpF3R2″XylT

The enzymatic reactions were performed in a final reaction volume of 100 mL containing 10 μg of purified recombinant EpF3R2″XylT proteins, 1 mM DTT, 100 mM Tris-HCl (pH 7.5), 0.5 mM substrates, and 4 mM UDP-glucose/UDP-rhamnose/UDP-xylose, and incubated at 30 °C for 1 h. The authentic flavonoid standards and UDP-glycose were purchased from the Shanghai Yuanye Bio-Technology Co., Ltd., Shanghai, China. The reactions were terminated via the addition of 100 mL of ice-cold methanol, followed by centrifugation at 14,000 rpm for 10 min. The supernatant of the reaction mixture was subjected to ACQUITY UPLC (UPLC I-class; Waters, Milford, MA, USA) with an ACQUITY UPLC BEH C18 column (2.1 × 100 mm. 1.7 μm; Waters, Milford, MA, USA). The mobile phase consisted of water (eluent A) and 100% acetonitrile (eluent B). The UPLC analysis was conducted with a flow rate of 0.3 mL·min^−1^ and the effluent was monitored at a 270 nm wavelength. The elution program was as follows: 

8-prenylflavonol glycosides: Starting with 5% eluent B, linear gradients of 5–27% B for 0–8 min, 27–80% B for 8–20 min, 80–100% B for 20–22 min, and 100% B for 23–24 min. 

Flavonols: Starting with 5% eluent B, linear gradients of 5–60% B for 0–8 min, 60–100% B for 8–16 min, 80–100% B for 16–17 min, and 100% B for 17–18 min.

Flavonol glycosides: Starting with 5% eluent B, linear gradients of 5–40% B for 0–10 min, 40–100% B for 10–12 min, and 100% B for 12–15 min.

LC–MS/MS analysis was performed on Waters ACQUITY UPLC I-Class/Xevo G2-XS QTOF (Waters, Milford, MA, USA) with a ZORBAX Eclipse Plus C18 (3.0 mm × 155 mm, 1.8 μm) at 32 °C. The mobile phase consisted of 0.1% formic acid (A) and 100% acetonitrile (B). Samples were run with a gradient elution as follows: 5% B for 0–1 min, 5–30% B for 1–8 min, 30–40% B for 8–12 min, 40–95% B for 12–16 min, 95–100% B for 16–17 min, and 100% B for 17–21 min. The operating conditions were as follows: A flow rate of 0.4 mL·min^−1^ with positive ion ESI mode, capillary voltage at 2.5 kV, cone voltage at 25 V, and desolvation gas flow at 1000 L·h^−1^. The mass-to-charge ratio was scanned from 200 to 1500 *m*/*z*.

### 4.6. NMR Analysis of Enzymatic Product

The preparative enzymatic reactions were performed in a final reaction volume of 20 mL containing 1.2 mg of purified recombinant EpF3R2″XylT proteins, 1 mM DTT, 100 mM Tris-HCl (pH 7.5), 0.5 mM icariin, and 4 mM UDP-xylose, and incubated at 30 °C for 24 h. The reactions were terminated via the addition of 20 mL of ice-cold methanol. The product was concentrated by rotary evaporation and dissolved in methanol, followed by purification with reverse-phase semi-preparative HPLC on an Innova ODS-2 column (250 × 4.6 mm. 5 μm; Agela Technologies, Beijing, China) at 35 °C. Approximately 1 mg of the prepared product was evaporated to dryness under N_2_ gas, dissolved in dimethyl sulfoxide-*d*_6_, and analyzed through ^1^H NMR, ^13^C NMR, HSQC, and NOESY with spectroscopic data analysis on a Bruker 600 spectrometer (Bruker, Rheinstetten, Germany).

The spectroscopic data of the prepared products are as follows:

Epimedin B (**1a**). ^1^H NMR (600 MHz, DMSO-*d*_6_) δ_H_ 7.83 (2H, m, H-2′/6′), 7.13 (2H, m, H-3′/5′), 3.00–5.50 (protons in rhamnose, glucose and xylose), 4.22 (1H, d, *J* = 7.0 Hz, H-1⁗), 4.09 (1H, m, H-2″), 3.85 (3H, s, OCH_3_), 1.67 (3H, s, H-15), 1.60 (3H, s, H-14). ^13^C NMR (151 MHz, DMSO-*d*_6_) δ_C_ 176.4 (C-4), 161.6 (C-4′), 161.3 (C-7), 155.1 (C-2), 154.8 (C-9), 135.9 (C-3), 130.6 (C-13), 121.0 (C-2′/6′), 124.6 (C-1′), 124.2 (C-12), 114.5 (C-3′/5′), 109.8 (C-8), 106.5 (C-10), 100.0 (C-1‴), 99.8 (C-6), 81.2 (C-2″), 77.3 (C-5‴), 76.6 (C-3‴), 73.8 (C-2⁗), 70.7 (C-4⁗), 70.2 (C-5″), 69.9 (C-4‴), 69.6 (C-3″), 66.2 (C-5⁗), 61.0 (C-6‴), 55.9 (OCH_3_), 26.0 (C-14), 21.9 (C-11), 18.2 (C-15).

### 4.7. Enzyme Kinetics of Recombinant EpF3R2″XylT

To determine the optimal reaction conditions for recombinant EpF3R2″XylT, a serial enzyme assay was conducted with UDP-xylose as a donor and icariin as an acceptor, each with three biological replicates. The enzymatic reaction was performed with 10 μg of recombinant EpF3R2″XylT, 10 mM DTT, 0.5 mM substrates, and 4 mM UDP-xylose, in a final volume of 50 μL. The reaction was terminated by adding an equal volume of ice-cold methanol. Samples were centrifuged at 14,000 rpm for 10 min and analyzed by UPLC. To determine the optimal reaction temperature, the reaction mixture was incubated at various temperatures ranging from 20 °C to 50 °C. To determine the optimal pH, the enzyme reaction was conducted in 100 mM Tris-HCl buffer from pH 4.5 to pH 10.5. In addition, the optimal incubation time was determined at 30 °C at multiple time points (1, 3, 6, 9, 12, 18, 24, and 36 h). 

For kinetic analysis, purified EpF3R2″XylT recombinant proteins (10 μg) were added to reaction mixtures containing 10 mM DTT, 100 mM Tris-HCl (pH 7.5), and 4 mM UDP-xylose in a final volume of 50 μL. The concentration of tested icariin ranged from 100 to 500 μM (100, 200, 300, and 500 μM). After incubation at 30 °C for 1 h, an equal volume of ice-cold methanol was added to terminate the reaction, followed by centrifugation at 14,000 rpm for 10 min. The reaction products were analyzed by UPLC. The software GraphPad Prism version 7.0.0 for Windows (GraphPad Software, San Diego, CA, USA, www.graphpad.com) was used to calculate kinetic parameters *K_m_*.

### 4.8. Bioinformatics Analyses of EpF3R2″XylT

The protein sequences of EpF3R2″XylT and previously characterized GTs involved in flavonoid biosynthesis (Table S1) were used for the sequence alignment and construction of the phylogenetic tree. The protein sequences were aligned with the ClustalX v2.0 program [29], and the phylogenetic tree was constructed with MEGA 11 software [30] based on the Maximum likelihood (ML) method with 1000 bootstrap replicates. 

The MEME online program (http://meme-suite.org/, accessed on 3 May 2022) was used to analyze the conservative motifs of EpF3R2″XylT and reported plant flavonoid GT protein sequences (Table S2) [31]. The maximum number of motifs was set to 15, and other parameters were default. The conserved motifs chart was drawn by the TBtools program [32].

The crystal structure of OsGT91C1 (Protein Data Bank code: 7erx.1) was used as a template to build the homology model of EpF3R2″XylT with the SWISS-MODEL server at http://swissmodel.expasy.org, accessed on 5 May 2022 [33]. Maestro software (Schrödinger, LLC, New York, NY, USA) was used to dock UDP-xylose (Compound CID: 19235) as the sugar donor and icariin (Compound CID: 5318997) as the acceptor for the model of EpF3R2″XylT. The model was visualized using the Pymol molecular graphics system (http://www. pymol.org, accessed on 8 May 2022).

### 4.9. Transient Expression of EpF3R2″XylT in Tobacco

*EpF3R2″XylT* was cloned into the pCAMBIA1302 vector by using the LanGene Seamless Cloning Assembly Kit (LANY, Beijing, China) with specific primers (Table S2). The construct and empty pCAMBIA1302 vector (negative control) were transformed into GV3101 (Biomed, Beijing, China). The single positive clone of pCAMBIA1302-*EpF3R2″XylT* and empty pCAMBIA1302 vector was incubated in LB media at 28 °C until the OD_600_ reached 0.6–0.8, the harvested cells were resuspended in AS buffer (pH 5.6, 10 mM MES, 10 mM Na_3_PO_4_, and 100 μM acetosyringone) with the final OD_600_ of 0.6–0.8, and the suspension was infiltrated into leaves of 6 week-old *Nicotiana Benthamiana* with a needle-less syringe after 2 h at room temperature. After two days, the infiltrated leaves were observed under a confocal laser scanning microscope (LEICA TCS SP8, Wetzlar, Germany). 

### 4.10. Extraction of Crude EpF3R2″XylT Protein from Infiltrated Tobacco Leaves

*EpF3R2″XylT* was cloned into the entry vector pENTR/D-TOPO (Invitrogen, Waltham, CA, USA) and then was inserted into the pK7WG2D.1 vector (Invitrogen, CA, USA) using the LR ClonaseTM II Enzyme (Invitrogen, Waltham, CA, USA). The *EpF3R2″XylT* construct and empty vector pK7WG2D.1 (negative control) were transformed into GV3101 (Biomed, Beijing, China) and infiltrated the leaves of *N. benthamiana*. The infiltrated leaves were collected and ground in liquid N_2_ after 3 days. Approximately 1 g of the sample powder was extracted with 4 mL 50 mM Tris-HCl (pH 8.0), 1 mM DTT, 5% (*w/v*) polyvinylpolypyrrolidone and sonicated on ice for 2 min, followed by centrifugation at 14,000 rpm for 15 min at 4 °C. Then the supernatant was filtered through Amicon Ultra-15 Ultra 10K (Millipore, MA, USA) and equilibrated with 50 mM Tris-HCl (pH 8.0), 1 mM DTT, 0.01% bovine serum albumin, and 5 mM b-mercaptoethanol. The concentrate was collected in a pre-cooled tube and used as the crude protein. The enzymatic reactions were performed in a final reaction volume of 100 μL containing appropriate crude EpF3R2″XylT protein, 1 mM DTT, 50 mM Tris-HCl (pH 8.0), 0.01% bovine serum albumin, 5 mM b-mercaptoethanol, 0.5 mM icariin, and 4 mM UDP-xylose, and incubated at 30 °C for 1 h. The reactions were terminated by the addition of 100 μL of ice-cold methanol, followed by centrifugation at 14,000 rpm for 10 min.

### 4.11. Accession Number

The nucleotide sequences of EpF3R2″XylT are available in the GenBank database under accession number ON569258.

## Figures and Tables

**Table 1:** Enzymatic kinetic parameters of recombinant EpF3R2″XylT proteins towards different flavonol substrates with UDP-xylose as donor.

GTs Substrates	*K_m_* (Μm)	*K_cat_* (S^−1^)	*K_cat_/K_m_* (S^–1^ Μm^–1^)
Icariin	75.96 ± 11.91	37.31	0.49
Baohuoside I	113.15 ± 37.60	16.64	0.15
Baohuoside II	123.97 ± 16.45	15.71	0.13

## Data Availability

All the raw data are available at the National Center for Biotechnology Information (NCBI) under project PRJNA747870. The genome database of *Epimedium pubescens* wass uploaded to the National Genomics Data Center (NGDC, https://bigd.big.ac.cn/, accessed on 20 October 2021) under BioProject PRJCA006303.

## Supplementary Materials

The following supporting information can be downloaded at: https://www.mdpi.com/article/10.3390/ijms232416050/s1, Figure S1: Multiple sequence alignment of EpF3R2″XylT and other known plant flavonoid GT proteins; Figure S2: Agarose gel electrophoresis analysis of DNA fragments amplified by PCR; Figure S3: SDS-PAGE of recombinant EpF3R2″XylT proteins; Figure S4: Chemical structures of flavonol substrates used in the enzymatic assays of recombinant EpF3R2″XylT proteins; Figure S5: Complete UPLC/ESI-MS spectra of flavonoid substrates and products in the enzymatic assays of recombinant EpF3R2″XylT proteins; Figure S6: The UPLC chromatographs of reaction catalyzed by recombinant EpF3R2″XylT proteins with prenylflavonols as substrates, which showed no activity; Figure S7: The UPLC chromatographs of reaction catalyzed by recombinant EpF3R2″XylT proteins with flavonols as substrates, which showed no activity; Figure S8: The UPLC chromatographs of reaction catalyzed by recombinant EpF3R2″XylT proteins with flavonol glycosides as substrates, which showed no activity; Figure S9: ^1^H NMR spectrum and ^13^C NMR spectrum of 1a in DMSO-*d*_6_; Figure S10: ^1^H-^13^C HSQC spectrum for 1a; Figure S11: ^1^H-^1^H NOESY spectrum for 1a. Table S1: GTs used for phylogenetic tree; Table S2: Primers used in this study; Table S3: Flavonoids used in the assays of EpF3R2″XylT enzymatic activity. Table S4: Mass spectrometry of product of EpF3R2″XylT enzymatic activity; Sequences of EpF3R2″XylT.
