# Supplementary material for: A Novel 3-O-rhamnoside: 2″-O-xylosyltransferase Responsible for Terminal Modification of Prenylflavonol Glycosides in Epimedium pubescens Maxim
Source: Int J Mol Sci. 2022 Dec 16;23(24):16050. doi: 10.3390/ijms232416050 (PMC9786081; doi:10.3390/ijms232416050)
Supplement: Supplementary file 1 [file ijms-23-16050-s001.zip › ijms-2012114-supplementary.pdf]

Supplementary Information

A Novel 3-*O*-rhamnoside: 2″-*O*-xylosyltransferase Responsible for Terminal Modification of Prenylflavonol Glycosides in *Epimedium pubescens* Maxim.

Yu Yao [1], Jiajun Gu [1,2], Yanjiao Luo [1], Yixin Zhang [1], Yuanyue Wang [1], Yongzhen Pang [3], Shangang Jia [4], Chaoqun Xu [1], Doudou Li [1], Fengmei Suo [1], Guoan Shen [1,*] and Baolin Guo [1,*]

1 Key Laboratory of Bioactive Substances and Resources Utilization of Chinese Herbal Medicines, Ministry of Education, Institute of Medicinal Plant Development, Chinese Academy of Medical Sciences, Peking Union Medical College, Beijing 100193, China
2 School of Chinese Materia Medica, Tianjin University of Traditional Chinese Medicine, Tianjin 301617, China
3 Institute of Animal Sciences, The Chinese Academy of Agricultural Sciences, Beijing 100193, China
4 College of Grassland Science and Technology, China Agricultural University, Beijing 100193, China
* Correspondence: gashen@implad.ac.cn (G.S.); blguo@implad.ac.cn (B.G.); Tel.: +86-10-57833172 (G.S. & B.G.)

1 Supplementary Figure Legends

Figure S1. Multiple sequence alignment of EpF3R2″XylT and other known plant flavonoid GT proteins. PSPG (the plant secondary product glycosyltransferase) domain was marked with red box.

Figure S2. Agarose gel electrophoresis analysis of DNA fragments amplified by PCR. Lane 1: DNA fragments amplified by first round PCR; Lane 2: DNA fragments amplified by second round PCR.

Figure S3. SDS-PAGE of recombinant EpF3R2″XylT proteins. Lane M: Protein ladder; Lane 1: Purified recombinant proteins.

Figure S4. Chemical structures of flavonol substrates used in the enzymatic assays of recombinant EpF3R2″XylT proteins.

Figure S5. Complete UPLC/ESI-MS spectra of flavonoid substrates and products in the enzymatic assays of recombinant EpF3R2″XylT proteins.

Figure S6. The UPLC chromatographs of reaction catalyzed by recombinant EpF3R2″XylT proteins with prenylflavonols as substrates, which showed no activity. Glc: glucose; Rha: rhamnose; Xyl: xylose. Prenylflavonols were marked with red arrow.

Figure S7. The UPLC chromatographs of reaction catalyzed by recombinant EpF3R2″XylT proteins with flavonols as substrates, which showed no activity. Glc: glucose; Rha: rhamnose; Xyl: xylose. Flavonols was marked with red arrow.

Figure S8. The UPLC chromatographs of reaction catalyzed by recombinant EpF3R2″XylT proteins with flavonol glycosides as substrates, which showed no activity. Glc: glucose; Rha: rhamnose; Xyl: xylose. Flavonol glycosides was marked with red arrow.

Figure S9. ^{1}H NMR spectrum and ^{13}C NMR spectrum of **1a** in DMSO-d_6. (a) ^{1}H NMR spectrum of **1a**. (b) ^{13}C NMR spectrum of **1a**.

Figure S10. ^{1}H-^{13}C HSQC spectrum for **1a**. The red arrow highlighted cross-peaks between H-2″ (δ_H 4.09) and C-2″ (δ_C 81.21).

Figure S11. ^{1}H-^{1}H NOESY spectrum for **1a**. The red arrow highlighted cross-peaks between H-2″ (δ_H 4.09) and H-1″″ (δ_H 4.22). The green line on the compound structure shows the relationship between the hydrogen bonds.

Figure S1

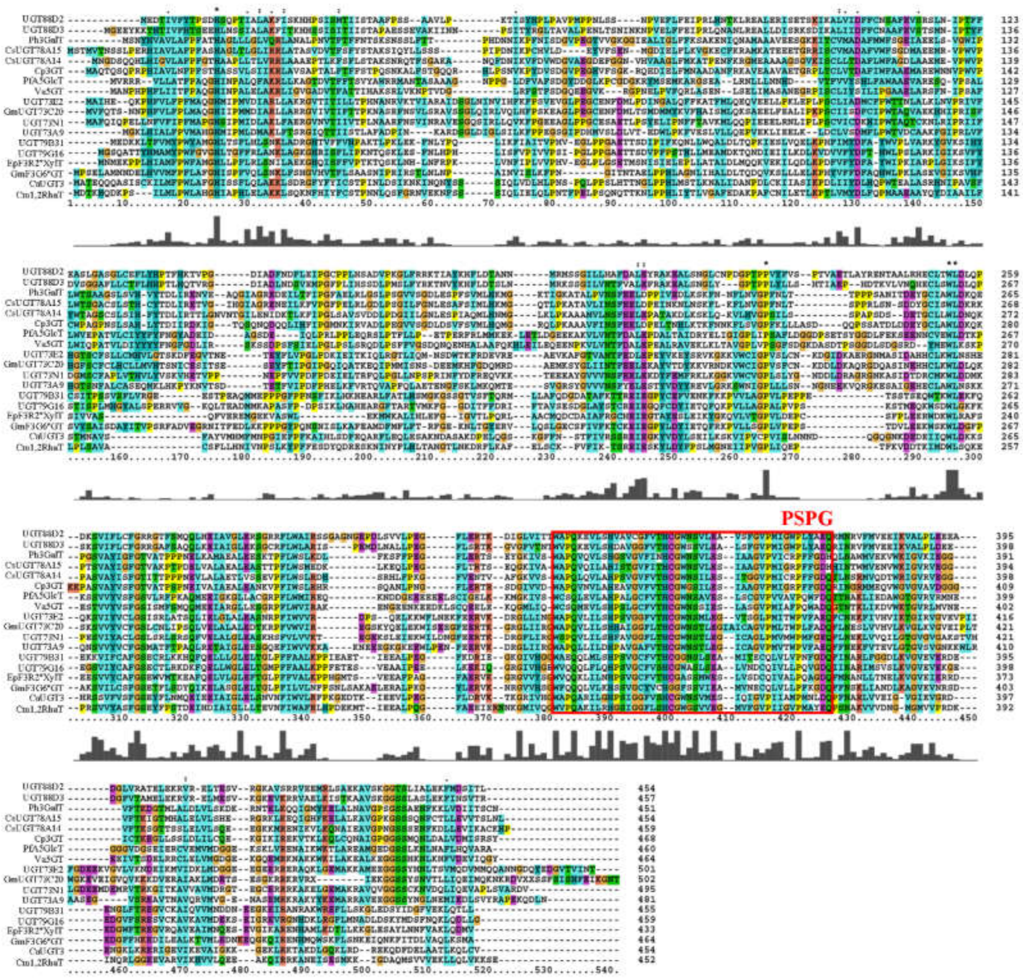

Figure S2

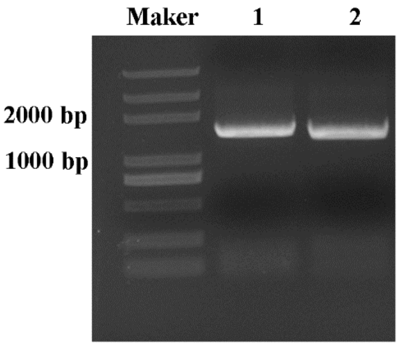

Figure S3

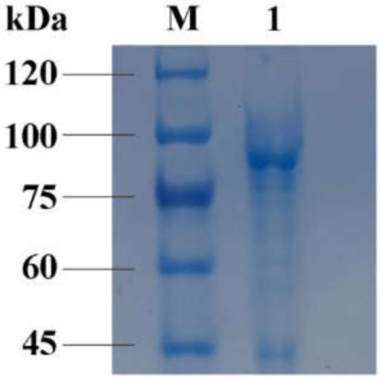

Figure S4

Flavonols

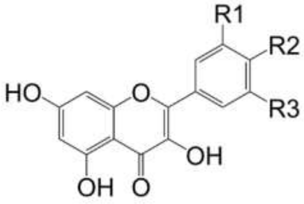

R1=H, R2=OH, R3=H, Kaempferol
R1=OH, R2=OH, R3=H, Quercetin
R1=OH, R2=OH, R3=OH, Myricetin
R1=H, R2=OCH_3, R3=H, Kaempferide
R1=OH, R2=OCH_3, R3=H, Tamarixetin
R1=OH, R2=OCH_3, R3=OH, Mearnsetin
R1=OCH_3, R2=OH, R3=H, Isorhamnetin

Favonol glycosides

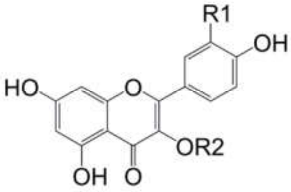

R1= H, R2=Glc, Kaempferide-3-O-glucoside
R1= H, R2=Rha, Kaempferide-3-O-rhamnoside
R1= H, R2=Gal, Kaempferide-3-O-galactoside
R1= OH, R2=Glc, Quercetin-3-O-glucoside
R1=OH, R2=Rha, Quercetin-3-O-rhamnoside
R1=OH, R2=Gal, Quercetin-3-O-galactoside
R1= OCH_3, R2=Glc, Isorhamnetin-3-O-glucoside
R1= OCH_3, R2=Rha, Isorhamnetin-3-O-rhamnoside

8-prenylflavonol glycosides

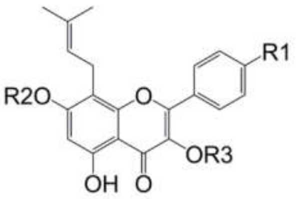

R1= OCH_3, R2=Glc, R3=Rha, Icaritin
R1= OCH_3, R2=H, R3=Rha, Baohuoside I
R1= OH, R2=H, R3=Rha, Baohuoside II
R1= OH, R2=Glc, R3=Rha, Epimedoside A
R1= OCH_3, R2=Glc, R3=H, Icariside I
R1= OCH_3, R2=Glc, R3=Rha^2-Glc, Epimedin A
R1= OCH_3, R2=Glc, R3=Rha^2-Xyl, Epimedin B
R1= OCH_3, R2=Glc, R3=Rha^2-Rha, Epimedin C

Figure S5

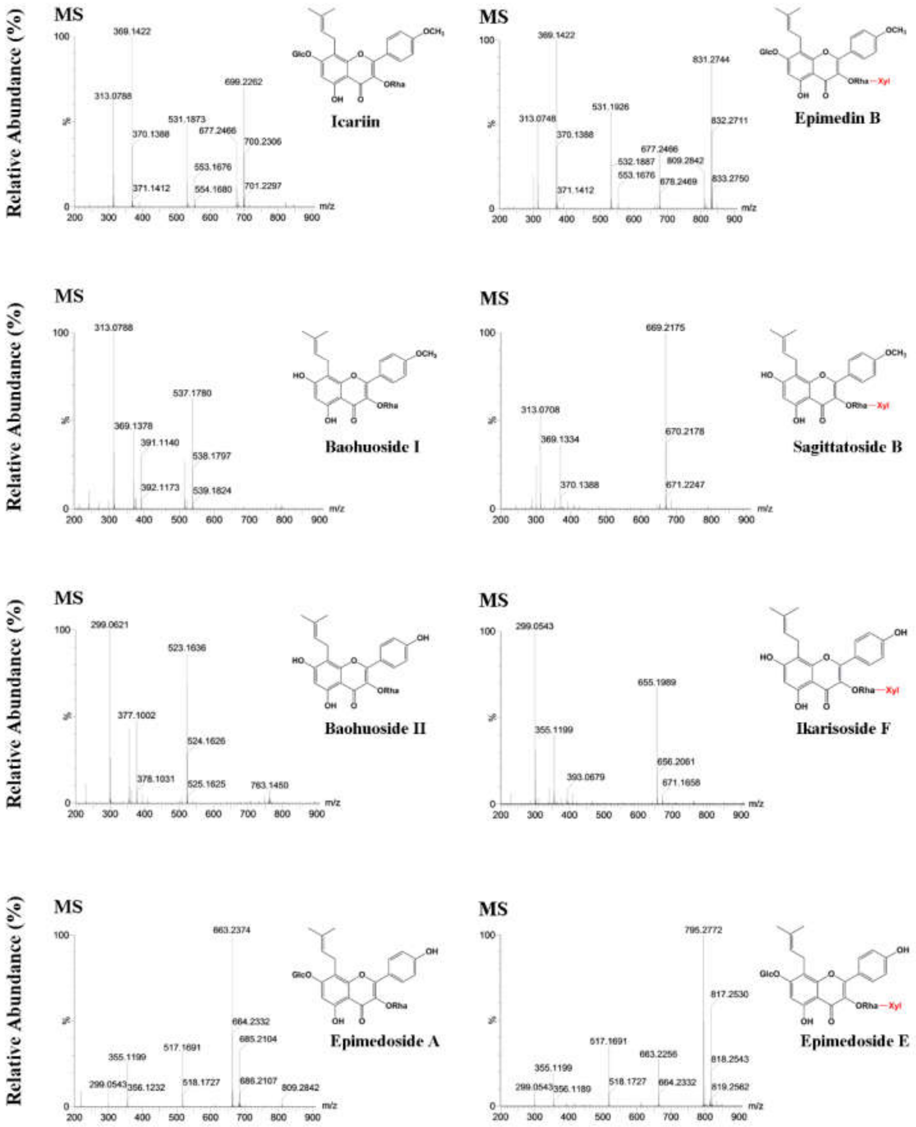
MS
Relative Abundance (%)
Icariin
369.1422
313.0788
370.1388
371.1412
531.1873
553.1676
554.1680
677.2466
699.2262
700.2306
701.2297
Epimedin B
369.1422
313.0748
370.1388
371.1412
531.1926
532.1887
553.1676
677.2466
678.2469
809.2842
831.2744
832.2711
833.2750
Baohuoside I
313.0788
369.1378
391.1140
392.1173
537.1780
538.1797
539.1824
Sagittatoside B
669.2175
313.0708
369.1334
370.1388
670.2178
671.2247
Baohuoside II
299.0621
377.1002
378.1031
523.1636
524.1626
525.1625
763.1450
Ikarisoside F
299.0543
355.1199
393.0679
655.1989
656.2061
671.1658
Epimedoside A
663.2374
355.1199
299.0543
356.1232
517.1691
518.1727
664.2332
685.2104
686.2107
809.2842
Epimedoside E
795.2772
355.1199
299.0543
356.1189
517.1691
518.1727
663.2256
664.2332
817.2530
818.2543
819.2562
m/z

Figure S6

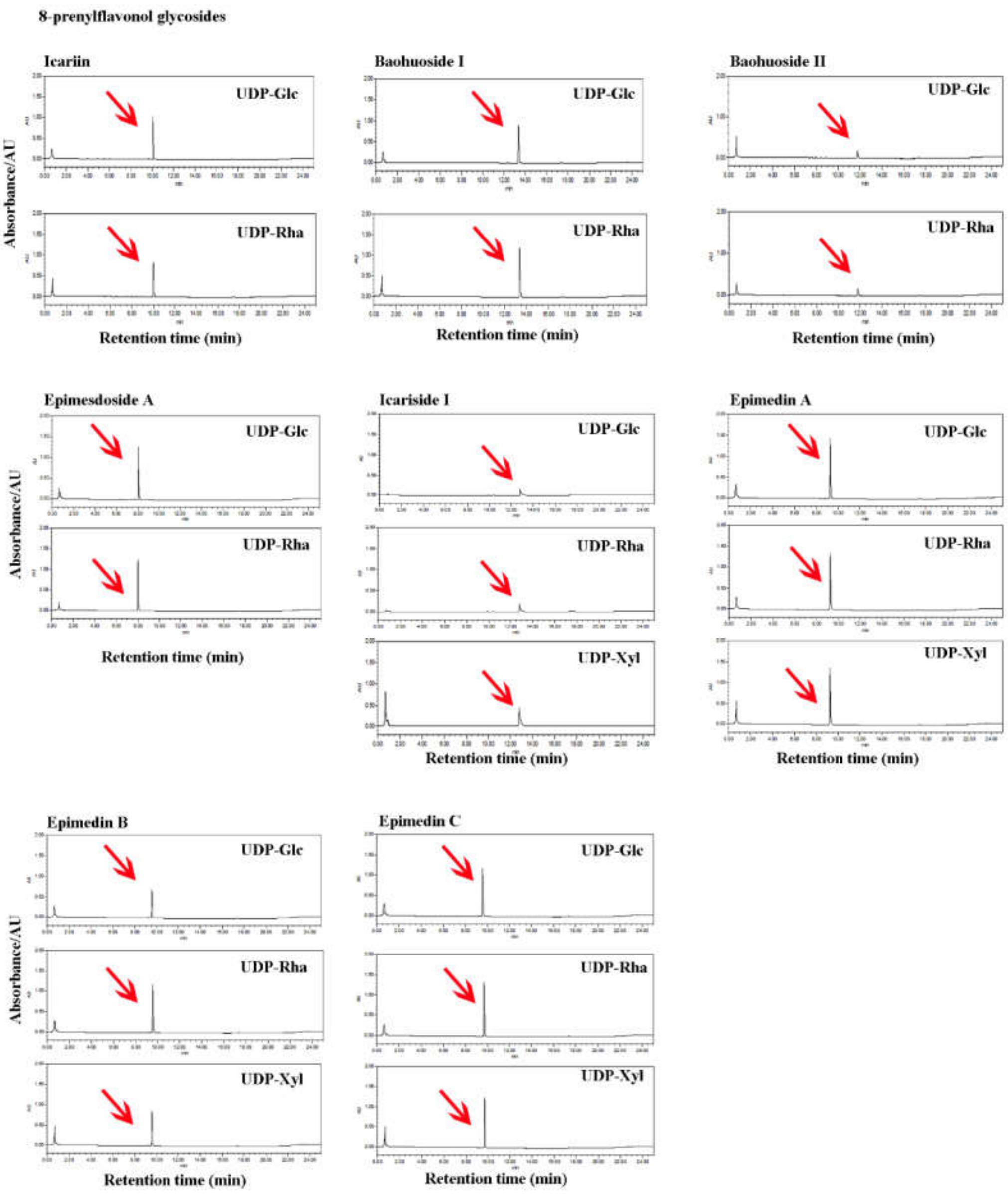
8-prenylflavonol glycosides
Icariin
UDP-Glc
UDP-Rha
Baohuoside I
UDP-Glc
UDP-Rha
Baohuoside II
UDP-Glc
UDP-Rha
Absorbance/AU
Retention time (min)
Retention time (min)
Retention time (min)
Epimesdoside A
UDP-Glc
UDP-Rha
Icariside I
UDP-Glc
UDP-Rha
UDP-Xyl
Epimedin A
UDP-Glc
UDP-Rha
UDP-Xyl
Absorbance/AU
Retention time (min)
Retention time (min)
Retention time (min)
Epimedin B
UDP-Glc
UDP-Rha
UDP-Xyl
Epimedin C
UDP-Glc
UDP-Rha
UDP-Xyl
Absorbance/AU
Retention time (min)
Retention time (min)

Figure S7

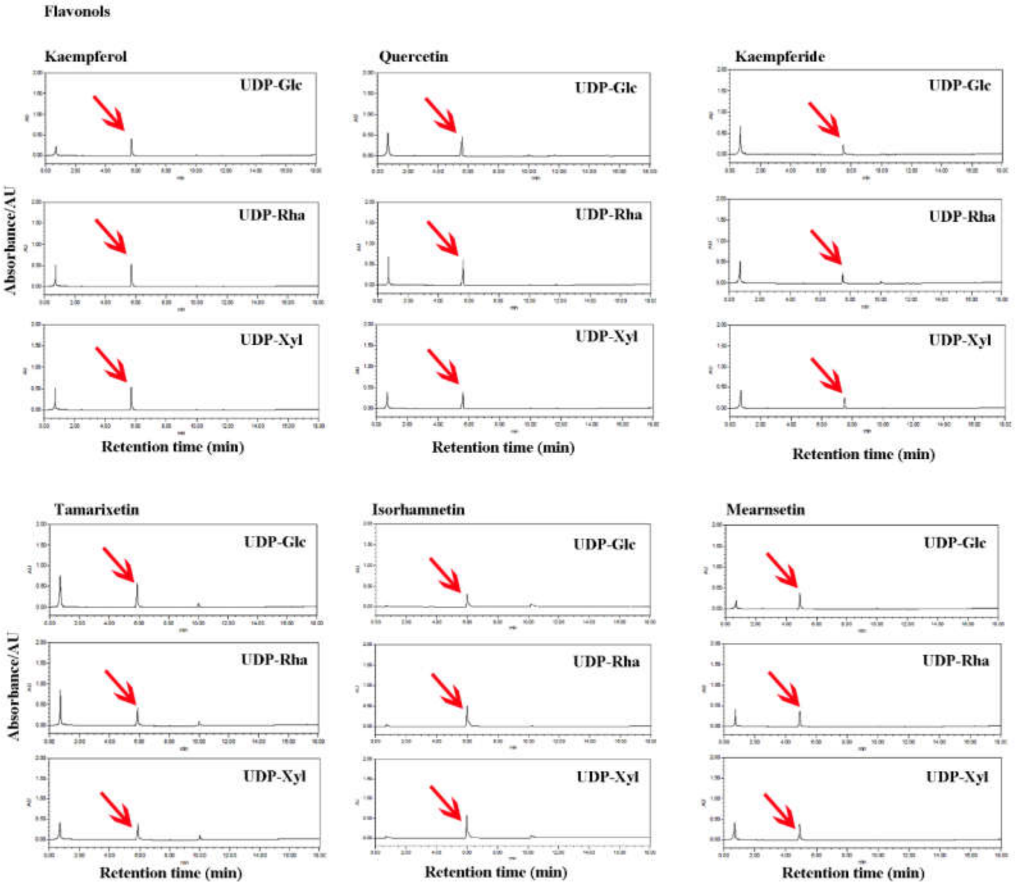
Flavonols
Kaempferol
Quercetin
Kaempferide
UDP-Glc
UDP-Rha
UDP-Xyl
Absorbance/AU
Retention time (min)
Tamarixetin
Isorhamnetin
Mearnsetin

Figure S8

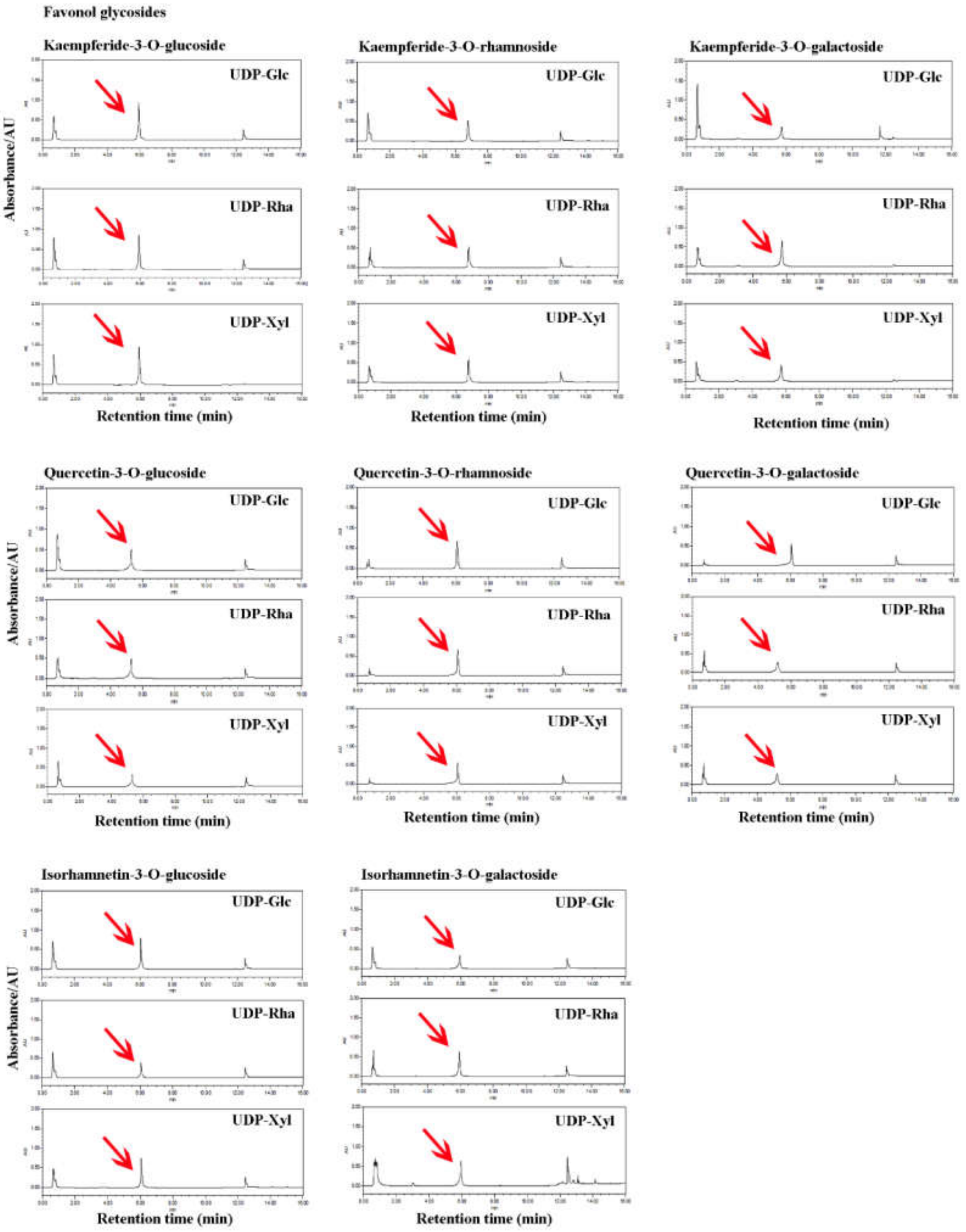

Favonol glycosides
Kaempferide-3-O-glucoside
Kaempferide-3-O-rhamnoside
Kaempferide-3-O-galactoside
Quercetin-3-O-glucoside
Quercetin-3-O-rhamnoside
Quercetin-3-O-galactoside
Isorhamnetin-3-O-glucoside
Isorhamnetin-3-O-galactoside
UDP-Glc
UDP-Rha
UDP-Xyl
Absorbance/AU
Retention time (min)

Figure S9

a

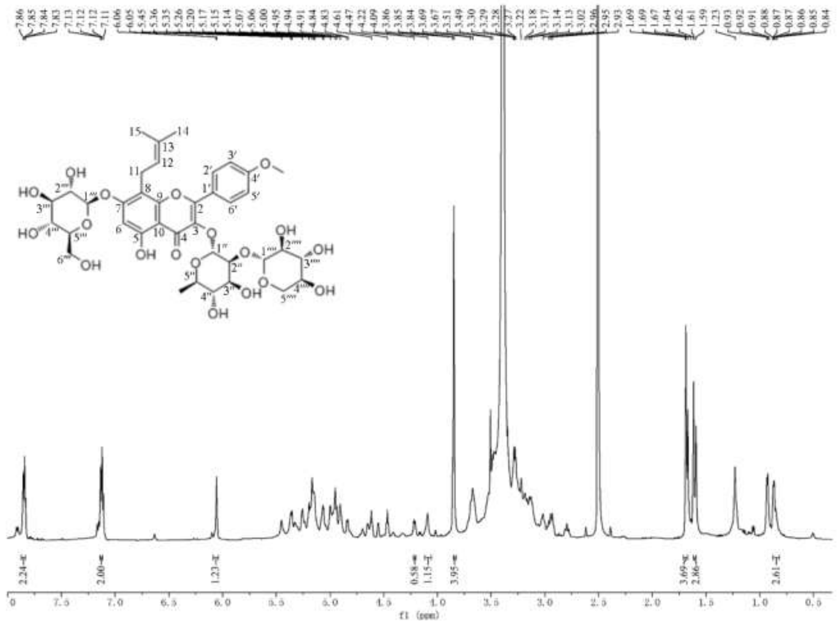

b

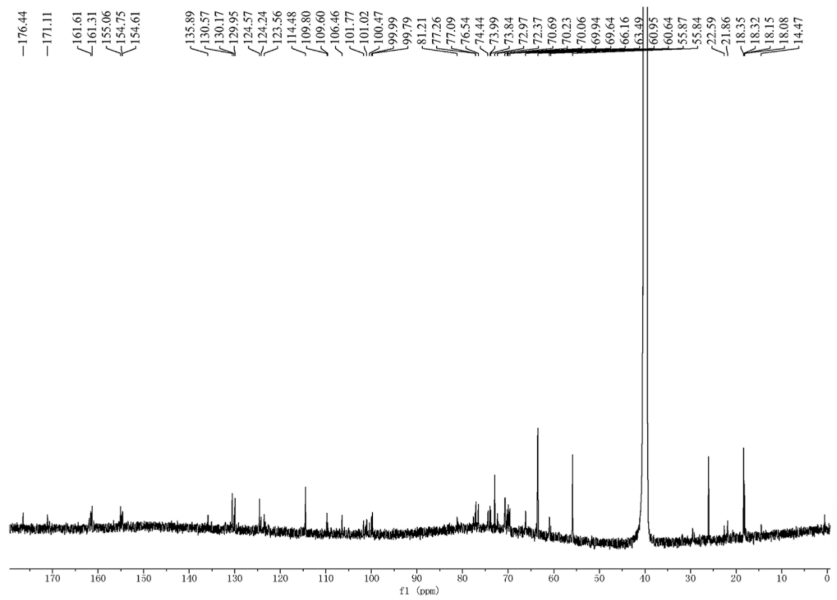

Figure S10

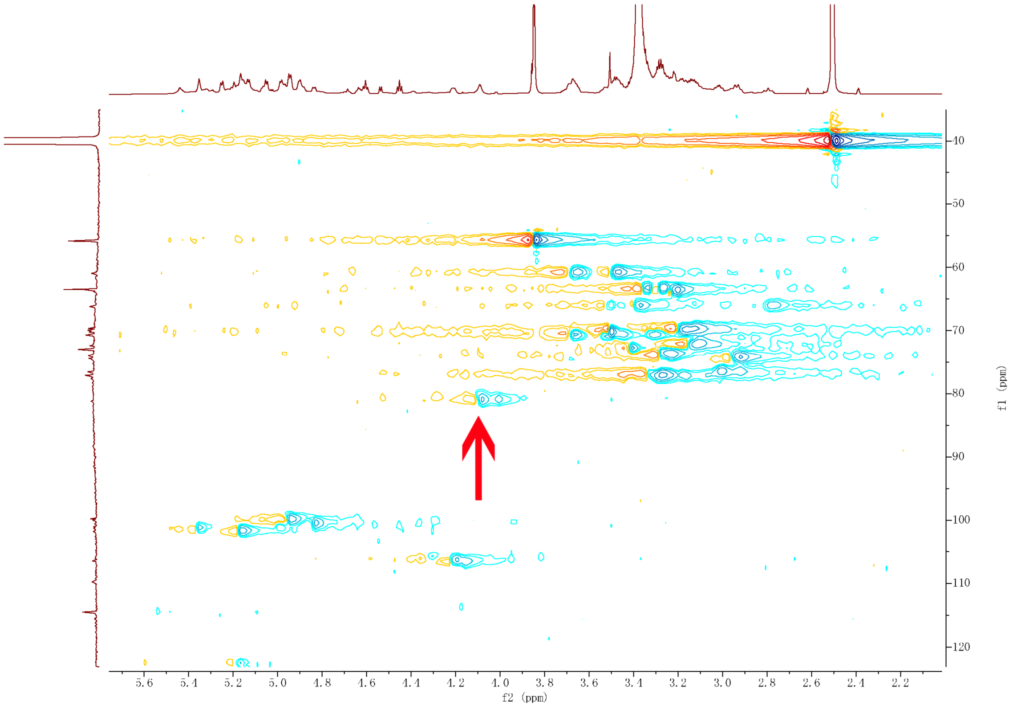

Figure S11

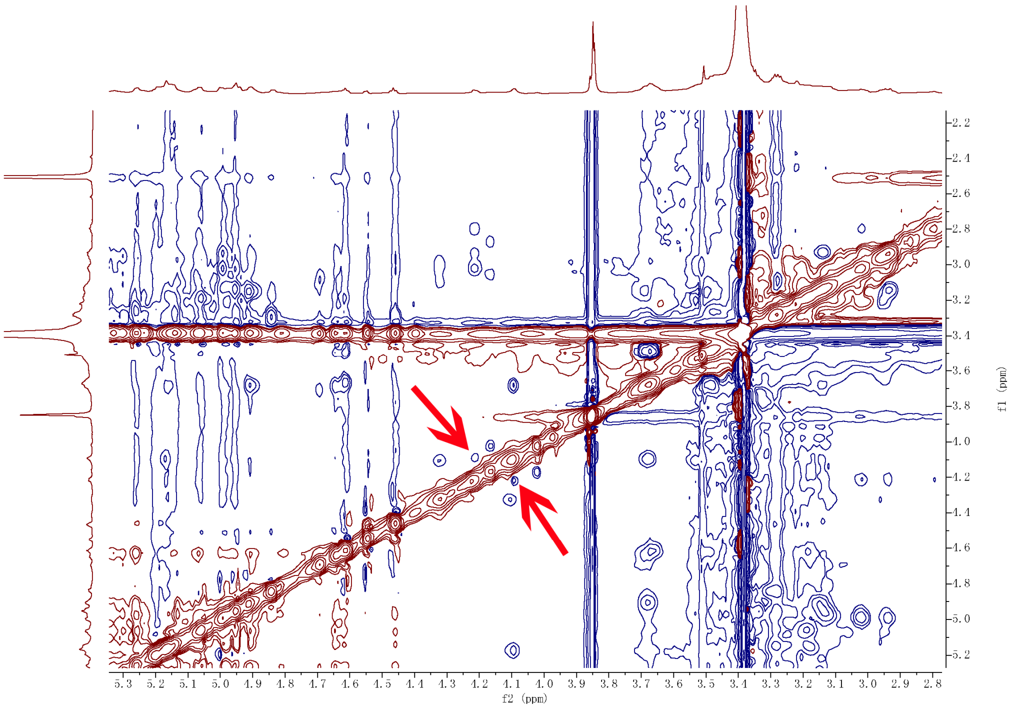

2 Supplementary Tables

Supplementary Table S1. GTs used for phylogenetic tree

Protein name	Length	Plant species	Accession ID	Modification site	Substrates
GmF3G6″Gt	464	*Glycine max*	NP_001345940.1	Glc-6"	Flavonol 3-*O*-glucoside
CaUGT3	454	*Catharanthus roseus*	AB443870	Glc-6"	Flavonol 3-*O*-glucoside
UGT79B31	455	*Petunia hybrida*	BBE29003.1	Glc-2"	Flavonol 3-*O*-glycoside
UGT79G16	459	*Ipomoea purpurea*	AB192315.1	Glc-2"	Anthocyanidin 3-*O*-glucoside
Cm1,2RhaT	452	*Citrus maxima*	AY048882	Glc-2"	Flavone 7-*O*-glucoside
AtUGT79B1	467	*Arabidopsis thaliana*	NP_200217.1	Glc-2"	Anthocyanidin 3-*O*-glucoside
UGT73A9 AmF7GlcT	481	*Antirrhinum majus*	BAG31950.1	7-OH	Flavone
UGT73E2 Am7GlcT	501	*Antirrhinum majus*	BAG16513.1	7-OH	Flavone
UGT73N1 Am7GlcT	495	*Antirrhinum majus*	BAG16514.1	7-OH	Flavone
GmUGT73C20	502	*Glycine max*	XP_003554403	7-OH	Isoflavone
Ph3GalT	451	*Petunia hybrida*	Q9SBQ8.1	3-OH	Flavonol
CsUGT78A14	459	*Camellia sinensis*	KP682360.1	3-OH	Flavonol
CsUGT78A15	454	*Camellia sinensis*	KP682361.1	3-OH	Flavonol
Cp3GT	468	*Citrus* x *paradisi*	ACS15351.1	3-OH	Flavonol
PfA5GlcT	460	*Perilla frutescens*	AHL68667	5-OH	Anthocyanidin 3-*O*-glucoside
Va5GT	464	*Vitis amurensis*	AHL68667.1	5-OH	Anthocyanidin
UGT88D2 LvC4'GlcT	454	*Linaria vulgaris*	BAE48240.1	4'-OH	Chalcone
UGT88D3 AmC4'GlcT	457	*Antirrhinum majus*	Q33DV3.1	4'-OH	Chalcone
EpF3R2″XylT	433	*Epimedium pubescens*	ON569258	Rha-2"	Prenylflavonol 3-*O*-rhamnoside

Supplementary Table S2. Primers used in this study

Gene ID	Primers	Sequences (5'-3')
EpF3R2″XylT	EpF3R2″XylT-F1	TTAGACCTGATAATCTCCAGTTG
(first round)	EpF3R2″XylT-R1	TAAATGTTGGATGGGTCACACAG
EpF3R2″XylT	EpF3R2″XylT-F2	ATGAACATGGAAAAGCCTCCTT
(second round)	EpF3R2″XylT-R2	TCAGACCATATCTTGCAGCTTTG
EpF3R2″XylT	EpF3R2″XylT-EcoRI	CGAGGGAAGGATTTCA<u>GAATTC</u>ATGAACATGGAAAAGCCTCCTT
(for pMAL-c2X)	EpF3R2″XylT-XbaI	CTTGCCTGCAGGTCGAC<u>TCTAGA</u>TCAGACCATATCTTGCAGCTTTG
EpF3R2″XylT	EpF3R2″XylT-NcoI	AGAACACGGGGGACTCTTGA<u>CCATGG</u>TAATGAACATGGAAAAGCCT
(for pCAMBIA1302)	EpF3R2″XylT-SpeI	GTGAAAAGTTCTTCTCCTTT<u>ACTAGT</u>GACCATATCTTGCAGCTTTG
EpF3R2″XylT	EpF3R2″XylT-qPCR-F	ATGACATCAGTAGAGGAAG
(qPCR)	EpF3R2″XylT-qPCR-R	GACAATCACTCACTAACG
Actin (qPCR)	Actin-qPCR-F	TCAGAAGGAAGAACATACAC
	Actin-qPCR-R	AAGAGAAGGACGGATAGC

Note: Enzyme digestion sites were underlined.

Supplementary Table S3. Flavonoids used in the assays of EpF3R2″XylT enzymatic activity

Substrate type	Substrate names	EpF3R2″XylT
**8-prenylflavonol glycosides**	Icaritin	++
	Baohuoside I	+
	Baohuoside II	+
	Epimedoside A	+
	Icariside I	-
	Epimedin A	-
	Epimedin B	-
	Epimedin C	-
**Flavonols**	Kaempferol	-
	Quercetin	-
	Kaempferide	-
	Tamarixetin	-
	Isorhamnetin	-
	Mearnsetin	-
**Favonol glycosides**	Kaempferide-3-*O*-glucoside	-
	Kaempferide-3-O-rhamnoside	-
	Kaempferide-3-*O*-galactoside	-
	Quercetin-3-*O*-glucoside	-
	Quercetin-3-*O*-rhamnoside	-
	Quercetin-3-*O*-galactoside	-
	Isorhamnetin-3-*O*-glucoside	-
	Isorhamnetin-3-*O*- galactoside	-

Note: + and - indicate if the enzyme activity is detectable or not, respectively.

Supplementary Table S4. Mass spectrometry of enzymatic product of EpF3R2″XylT

NO.	Compounds	Molecular formula	Molecular weight	$[M+H]^+$	Fragment ions
**1**	Icariin	$C_{33}H_{40}O_{15}$	676.7	677.2584	531.1926 $[M+H\text{-}Rha]^+$, 369.1378 $[M+H\text{-}Rha\text{-}Glc]^+$, 313.0708 $[M+H\text{-}Rha\text{-}Glc\text{-}C_4H_8]^+$
**1a**	Epimedin B	$C_{38}H_{48}O_{19}$	808.8	809.2971	677.2466 $[M+H\text{-}Xyl]^+$, 531.1873 $[M+H\text{-}Xyl\text{-}Rha]^+$, 369.1334 $[M+H\text{-}Xyl\text{-}Rha\text{-}Glc]^+$, 313.0708 $[M+H\text{-}Xyl\text{-}Rha\text{-}Glc\text{-}C_4H_8]^+$
**2**	Baohuoside I	$C_{27}H_{30}O_{10}$	514.5	515.1958	369.1422 $[M+H\text{-}Rha]^+$, 313.0708 $[M+H\text{-}Rha\text{-}C_4H_8]^+$
**2a**	Sagittatoside B	$C_{32}H_{38}O_{14}$	646.6	647.2325	515.1906 $[M+H\text{-}Rha]^+$, 369.1334 $[M+H\text{-}Rha\text{-}Glc]^+$, 288.2591 $[M+H\text{-}Rha\text{-}Glc\text{-}C_4H_8]^+$
**3**	Baohuoside II	$C_{26}H_{28}O_{10}$	500.5	501.1778	355.1241 $[M+H\text{-}Rha]^+$, 299.0543 $[M+H\text{-}Rha\text{-}C_4H_8]^+$
**3a**	Ikarisoside F	$C_{31}H_{36}O_{14}$	632.6	633.2184	501.1778 $[M+H\text{-}Xyl]^+$, 355.1199 $[M+H\text{-}Xyl\text{-}Rha]^+$, 299.0543 $[M+H\text{-}Xyl\text{-}Rha\text{-}C_4H_8]^+$
**4**	Epimedoside A	$C_{32}H_{38}O_{15}$	662.6	663.2374	517.1691 $[M+H\text{-}Rha]^+$, 355.1199 $[M+H\text{-}Rha\text{-}Glc]^+$, 299.0543 $[M+H\text{-}Rha\text{-}Glc\text{-}C_4H_8]^+$
**4a**	Epimedoside E	$C_{37}H_{46}O_{19}$	794.7	795.2772	663.2256 $[M+H\text{-}Xyl]^+$, 517.1691 $[M+H\text{-}Xyl\text{-}Rha]^+$, 355.1199 $[M+H\text{-}Xyl\text{-}Rha\text{-}Glc]^+$, 299.0543 $[M+H\text{-}Xyl\text{-}Rha\text{-}Glc\text{-}C_4H_8]^+$

3 Sequences of *EpF3R2''XylT*

3.1 DNA sequence

ATGAACATGGAAAAGCCTCCTTTACATATTGCTATGTTTCCATGGTTTGCCATGGGCCATCTGCTTCCCTTTCTCCGCCTTTC
AAACATCTTAGCAGAGAAAGGCCACCAAATCTCCTTTTTTGTACCCACAAAAACCCAGTCCAAGTTAAACCATCTCAATTTTC
GTCCCAAACTCGTCAATTTCATTCCCCTTGTTGTTCCTCATGTAGAAGGCCTTCCACTTGGTTCTGAAACCATGTCGAACATC
TCAATCGAACTTGAACCCCTCCTTGCAACTGCCTTAGACCTTATGCAACAAAAGGTTGAAAAAATTCTTCAAGATCTAAAACC
TGATTTTGTTTTCTACGACTTCGCCTACTGGATACCAAAAATTGCTCGTCCCCTTGGGATCAAGTCCATATTCTACTCAATTG
TGGTTGCATCACAATTTGTTGAACGTGAAATGGGCGAAAAGGTCGCATCTTGGCTTGAGAAAATGTGGAAAGCACTCATCCAT
CTTGAATTTGGGATTGGGGTAACATTGCCTCAAAGACTTGCTGCCTGCATGCAGGACTGCGATGCCATTGCCTTCAGAGGGTG
TCATGAAATCGAGGGAACAGCCTATGAATCCCTTGAGATAAAGTATGGGAAACAAGTACTCGTAACTGGTCCAGTTTTGGATG
AACCATGTAGTTTCCCTTTGGAAGAGCGTTGGGATAAGTGGTTAAGGGCATTTTCAGAGGAATCTGTAGTTTACTGTGCGTTT
GGGAGCGAGTGGGTTATGACTAAAGAAGCATTTCAGGAATTGGTCTTAGGTTTGGAGTTTACTGGATTGCCATTCTTTGTGGC
ACTTAAACCACCACATGGGATGACATCAGTAGAGGAAGCATTCCCGGCTGGGTTCGCGGAAAGGGTGAAGGGAAGAGGGGTTG
TTTATTCGGGCTGGGTACAACAGAAGCTCATCCTAAACCACCCATCGGTGGGATGTTTTGTGACCCATTGCGGGGCTTCGTCA
ATGTGGGAATCGTTAGTGAGTGATTGTCAGATAGTAGCCCTGCCACAAGCAGGGGATCAGTTTATGAATGCTAATTTGTTGAC
AAACGAACTCAAGGTTGGTGTGGAAATTGAGCGGAGGGATGAGGATGGGTGGTTCACAAGGGAGGGAGTGCGTCAGGCTGTTG
AGGCTATAATGAACCAAGAGAGCGAAGTTGGTATAAAAGCCAGGGAAAACCATGCTATGTTGAAGGATACTTTGTTGAAGAAA
GGACTAGAATCGGCTTACCTGAACAATTTCGTTGCAAAGCTGCAAGATATGGTCTGA

3.2 Protein sequence

MNMEKPPLHIAMFPWFAMGHLLPFLRLSNILAEKGHQISFFVPTKTQSKLNHLNFRPKLVNFIPLVVPHVEGLPLGSETMSNI
SIELEPLLATALDLMQQKVEKILQDLKPDFVFYDFAYWIPKIARPLGIKSIFYSIVVASQFVEREMGEKVASWLEKMWKALIH
LEFGIGVTLPQRLAACMQDCDAIAFRGCHEIEGTAYESLEIKYGKQVLVTGPVLDEPCSFPLEERWDKWLRAFSEESVVYCAF
GSEWVMTKEAFQELVLGLEFTGLPFFVALKPPHGMTSVEEAFPAGFAERVKGRGVVYSGWVQQKLILNHPSVGCFVTHCGASS
MWESLVSDCQIVALPQAGDQFMNANLLTNELKVGVEIERRDEDGWFTREGVRQAVEAIMNQESEVGIKARENHAMLKDTLLKK
GLESAYLNNFVAKLQDMV
